# Switchable CAR-T cells mediate remission in metastatic pancreatic ductal adenocarcinoma

**DOI:** 10.1136/gutjnl-2018-316595

**Published:** 2018-08-18

**Authors:** Deepak Raj, Ming-Hsin Yang, David Rodgers, Eric N Hampton, Julfa Begum, Arif Mustafa, Daniela Lorizio, Irene Garces, David Propper, James G Kench, H M Kocher, Travis S Young, Alexandra Aicher, Christopher Heeschen

**Affiliations:** 1 Stem Cells in Cancer and Ageing, Barts Cancer Institute (BCI), Queen Mary University of London, London, UK; 2 Biologics, California Institute for Biomedical Research, La Jolla, California, USA; 3 Biological Service Unit, Barts Cancer Institute, London, UK; 4 Cancer and Inflammation, Barts Cancer Institute, London, UK; 5 Department of Tissue Pathology and Diagnostic Oncology, Royal Prince Alfred Hospital, Camperdown, New South Wales, Australia; 6 Director of the Barts Pancreatic Cancer Tissue Bank, Barts Cancer Institute (BCI), Queen Mary University of London, London, UK; 7 School of Medical Sciences, University of New South Wales, Sydney, New South Wales, Australia

**Keywords:** pancreatic cancer, stem cells, immunotherapy, liver metastases

## Abstract

**Objective:**

Pancreatic ductal adenocarcinoma (PDAC) is a disease of unmet medical need. While immunotherapy with chimeric antigen receptor T (CAR-T) cells has shown much promise in haematological malignancies, their efficacy for solid tumours is challenged by the lack of tumour-specific antigens required to avoid on-target, off-tumour effects. Switchable CAR-T cells whereby activity of the CAR-T cell is controlled by dosage of a tumour antigen-specific recombinant Fab-based ‘switch’ to afford a fully tunable response may overcome this translational barrier.

**Design:**

In this present study, we have used conventional and switchable CAR-T cells to target the antigen HER2, which is upregulated on tumour cells, but also present at low levels on normal human tissue. We used patient-derived xenograft models derived from patients with stage IV PDAC that mimic the most aggressive features of PDAC, including severe liver and lung metastases.

**Results:**

Switchable CAR-T cells followed by administration of the switch directed against human epidermal growth factor receptor 2 (HER2)-induced complete remission in difficult-to-treat, patient-derived advanced pancreatic tumour models. Switchable HER2 CAR-T cells were as effective as conventional HER2 CAR-T cells in vivo testing a range of different CAR-T cell doses.

**Conclusion:**

These results suggest that a switchable CAR-T system is efficacious against aggressive and disseminated tumours derived from patients with advanced PDAC while affording the potential safety of a control switch.

**Significance of this study**

**What is already known on this subject?**

- Chimeric antigen receptor T (CAR-T) cell therapy has demonstrated remarkable clinical success in haematological diseases.
- CAR-T cell therapy for pancreatic cancer via targeting of HER2 has shown promising results in cell line-based models.
- Potential on-target off-tumour effects due to low-level HER2 expression in the lung and other tissues may cause dangerous toxicity in patients; a titratable CAR-T system may therefore offer potential for safety without compromising efficacy.

**What are the new findings?**

- Switchable CAR-T cells followed by administration of a Fab-based switch directed against HER2 are highly effective against difficult-to-treat, patient-derived advanced pancreatic tumours
- The switchable HER2 platform is as effective as conventional HER2 CAR-T cells across a range of different CAR-T cell doses in patient-derived xenograft models.
- Dosage of the HER2 switch elicited significant cytokine production from switchable CAR-T cells in vivo, suggesting that switchable CAR-T cell activity can be modulated in vivo by administration (or absence) of switch.
- The switchable HER2 platform is expected to be an attractive therapeutic option to control activation of CAR-T cells for antigens such as HER2, which is upregulated in tumours but shared with normal tissue.

**How might it impact on clinical practice in the foreseeable future?**

- Due to its titratability, the switchable CAR-T cell platform against HER2 bears potential to safely improve the outcome of patients with advanced pancreatic cancer.

## Introduction

Pancreatic ductal adenocarcinoma (PDAC) is the fourth most common cause of cancer-related deaths, with a 5-year survival rate of less than 10%. Due to a lack of early symptoms, the disease is mostly diagnosed at an advanced stage (stage IV), with less than 20% of patients presenting with localised and therefore resectable tumours.^1^ As current therapies for patients with advanced disease are merely able to extend survival by a few months, PDAC is considered a disease with an urgent and unmet medical need. It is now understood that inherent or rapidly evolving chemoresistance and subsequent relapse is driven by a subset of cells with stem cell-like properties.^2–4^ Any new treatment developed for PDAC must also efficiently target this cancer stem cell (CSC) population to achieve durable responses.^5^

Chimeric antigen receptor T cells (CAR-T) have shown tremendous success against CD19-expressing B cell leukaemia.^6 7^ In contrast, CAR-T cell therapy of solid tumours is hindered by several factors: (1) the stiff desmoplastic nature of the tumour microenvironment (TME), which creates a physical barrier to T cell entry,^8^ (2) T cell exhaustion and anergy caused by the immune-inhibitory TME,^9^ (3) the shared expression of tumour antigens on vital tissues, including the stem cell compartment of normal tissues and (4) in the case of PDAC, the aggressive nature of the disease as well as the fact that diagnosis is usually at a late stage with much tumour dissemination. Unlike the B cell antigen CD19, virtually all solid tumour antigens are expressed lowly but broadly across a variety of normal tissues, which has resulted in severe toxicities in the translation of CAR-T therapy to solid tumours in the clinic. For example, a clinical trial using carbonic anhydrase IV (CAIX) CAR-T cells for metastatic renal carcinoma had to be halted due to liver toxicity that was related to cross-reactivity to CAIX-positive bile duct epithelium.^10^ In another trial, a patient with metastatic colon carcinoma treated with a third-generation CAR targeting HER2 succumbed to respiratory failure caused by CAR-T cells recognising low levels of HER2 expressed on the lung epithelium.^11^

**Figure 1:**
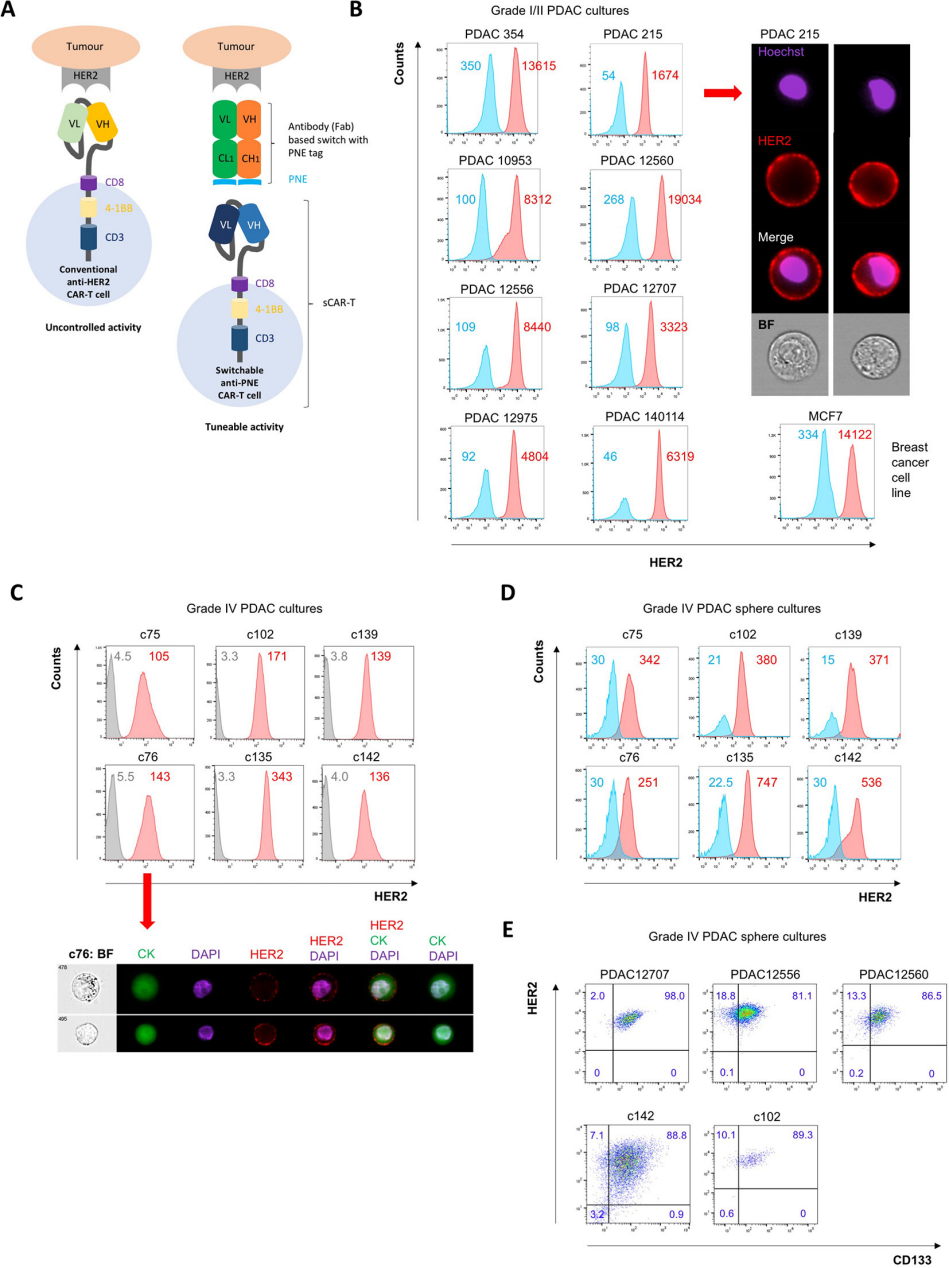
Expression of HER2 in PDAC cultures derived from patients with local stage I/II and advanced stage IV PDAC. (A) Schematic illustration of the switchable CAR-T system. (B) Expression of HER2 (red) in human primary stage I/II PDAC cultures versus secondary antibody only (blue) as control assessed by standard flow cytometry with mean fluorescence intensity (MFI) listed as inserts (left) and imaging flow cytometry using Hoechst 33 342 as nuclear stain and bright field (BF) to reveal cellular morphology (right); MCF7 breast cancer cells are used as positive control for HER2 expression. (C) Expression of HER2 in stage IV PDAC adherent cultures (red) and secondary antibody only (grey) as control by standard flow cytometry (upper). Imaging flow cytometry with dual staining for HER2 and pan-cytokeratin (CK) and DAPI used as nuclear stain (lower). (D) Expression of HER2 in CSC-enriched spheres from PDAC cultures and (E) costaining of HER2 with CD133 in CSC-enriched sphere cultures. Levels of expression are given as insert in percentage (%). CAR-T, chimeric antigen receptor T; CSC, cancer stem cell; PDAC, pancreatic ductal adenocarcinoma; PNE, peptide neoepitope; VH, variable domain, heavy chain; VL, variable domain, light chain.

Both CAIX and HER2 are safely targeted with monoclonal antibodies, whereby the mechanism of tumour killing is through antibody-dependent cell-mediated cytotoxicity (ADCC) and the activity of the canonical immune response can be safely controlled through dosing regimen.^12 13^ CAR-T cells, however, are a living drug that have enhanced T cell signalling through the CAR and proliferate extensively on antigen recognition; this exaggerated potency reduces the therapeutic window of targeting antigens that are expressed on healthy tissue, such as CAIX and HER2, and thus increases treatment-associated toxicity. Several efforts have thus been made to control the activity of CAR-T cells to enable the targeting of antigens with toxicity liabilities. We developed an antibody-based switchable CAR system whose activity can be titrated in vivo.^14 15^ These switchable CAR-T cells bind a specific peptide that is genetically engrafted onto a tumour-binding Fab molecule (figure 1A). The ‘switch’ acts as a bridge between target and effector cells and governs the antigen specificity, activation and ultimately tumour clearance. These switches have the advantages of the relatively short half-life of the Fab fragment and the use of peptide tags with limited immunogenicity.^16 17^ This versatile system has proven to be effective in preclinical models of leukaemia and breast cancer.^15–17^

HER2/ERBB2 has been reported to be expressed in PDAC,^18 19^ though estimates of its prevalence vary widely, from 10%^20^ to 60%.^21^ Here we show that a considerable subset of PDAC tumours express HER2 at detectable levels. We also show that HER2 is similarly expressed in the contained CSC and their differentiated progenies (non-CSC) and can be targeted by both healthy donor and patient-derived CAR-T cells. We use highly aggressive xenograft models newly generated from current stage IV PDAC patients and demonstrate that both local and disseminated disease can be efficiently targeted even when expressing relatively modest levels of HER2. Using dose titration of CAR-T cells, we demonstrate that switchable CAR-T cells afford comparable efficacy as conventional HER2 CAR-T cells in both orthotopic and metastatic PDAC models and that in vivo activity of switchable CAR-T cells (unlike conventional HER2 CAR-T cells) is contingent on administration of the switch. Our data suggest that the titratability of switchable CAR-T cells may allow safe targeting of HER2 in a clinical setting.

## Results

### HER2 expression in pancreatic CSC and non-CSC

Assessing HER2 expression on a tissue microarray, we found a range of expression on a panel of PDACs, with 6/16 samples showing high (3+) HER2 expression, 3/16 samples intermediate (2+) and 7/16 samples low (1+) (online supplementary table 1, online supplementary figure 1a). We next assessed HER2 expression in a panel of early passage PDAC cultures (<1 month in culture) isolated from surgical sections of primary tumours from patients with stage I/II PDAC. Such cultures are preferred over immortalised PDAC cell lines for several reasons: (1) primary cultures recapitulate the genetic heterogeneity of the original tumour and are more likely to predict an inpatient tumour response, (2) primary cultures, similar to the original tumour, contain a subpopulation of CSC that represent the root of the disease^22^ and (3) CAR-target antigen expression in primary cultures is a more accurate representation of the physiology of the original PDAC tumour rather than an artefact of long-term cell culture. We found a spectrum of HER2 expression in this panel, with some samples expressing HER2 at similar levels to the breast cancer line MCF-7 (figure 1B). Cultured human T cells, and freshly isolated primary human peripheral blood mononuclear cells (PBMC), none of whose subsets express HER2, were used as negative controls (online supplementary figure 1b).

10.1136/gutjnl-2018-316595.supp1 Supplementary data

We have now also generated various PDAC models from patients with stage IV PDAC, the most prevailing population of patients in the clinic (c75, c76, c102, c135, c139 and c142). The corresponding cultures harbour the most metastatic subpopulations as evidenced by their strong expression of CXCR4, high invasiveness and remarkable in vivo metastatic activity and thus are highly representative for advanced PDAC.^23^ We found HER2 expression in all cases, ranging from modest to very strong expression (figure 1C, online supplementary figure 1c).

CSCs represent the hierarchical root of PDAC, drive resistance to conventional therapy and are the principle cause of disease relapse. Thus, it is important to test any PDAC therapy for efficacy against pancreatic CSC. We therefore generated sphere cultures to enrich for CSC and probed sphere-derived cells for HER2 expression. We found similar levels of HER2 expression as in more differentiated adherent PDAC cultures, suggesting that HER2 is not altered in pancreatic CSC. HER2 expression on CSC was further confirmed by costaining for CD133 to specifically track CSC (figure 1D,E).

We next transduced cultures from stage I/II PDAC 215 and 354 and stage IV c76 to stably express GFP and luciferase for further analysis. Reprobing these cultures showed that HER2 expression was maintained after genetic modification, with highest expression on PDAC 215 (online supplementary figure 1d). We therefore used these primary cultures for further in vitro and in vivo work.

### HER2 CAR-T cells mediate cytotoxicity of HER2-expressing PDAC cultures

**Figure 2:**
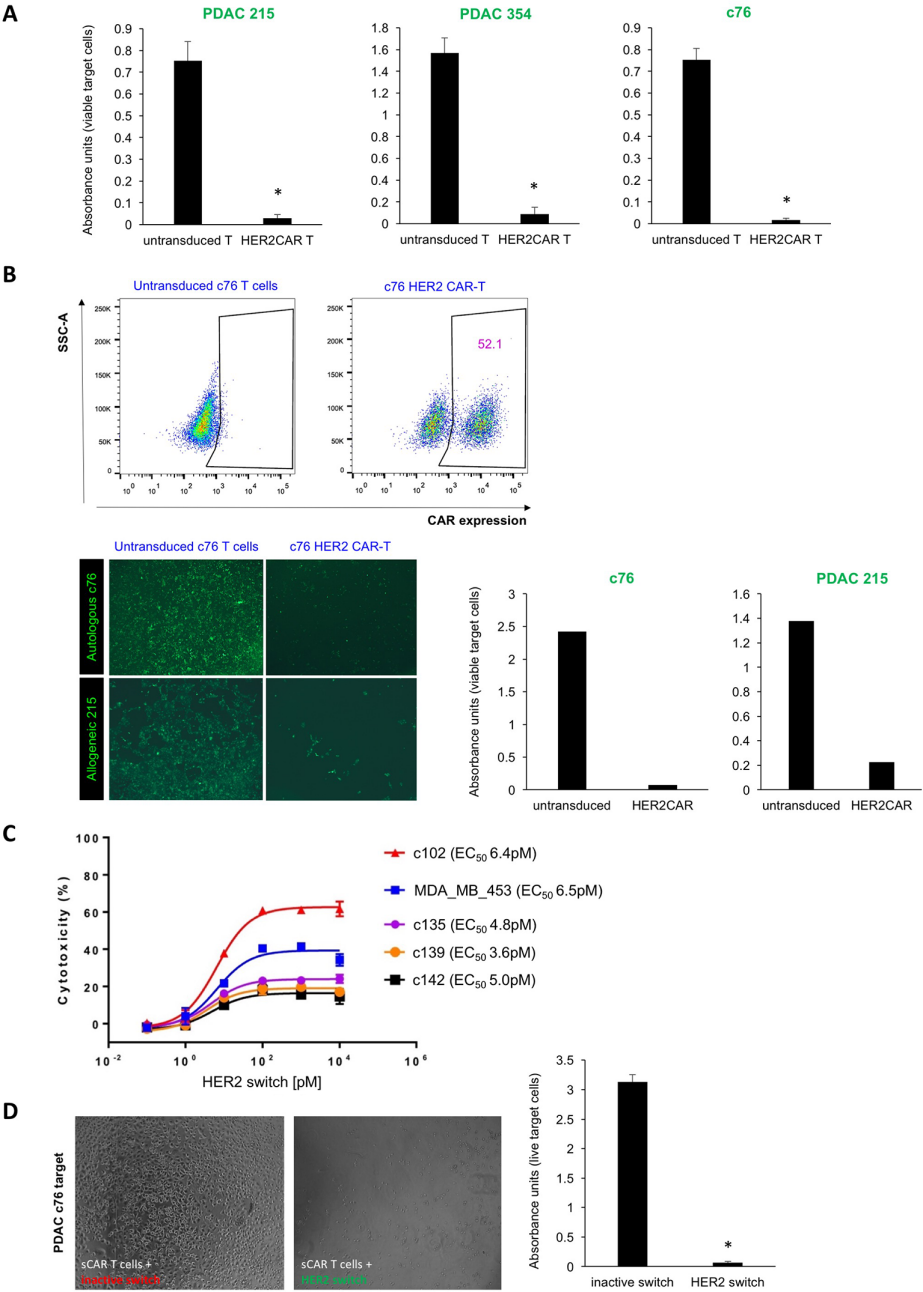
CAR-T cell-mediated cytotoxicity against bulk PDAC cells. (A) Lysis of a confluent layer of green fluorescence protein (GFP)-expressing PDAC target cell cultures in the presence of HER2 CAR-T cells or untransduced T cells (negative control). Quantification of target cell viability using WST-1 assays with absorbance units proportional to viable target cells; n=6, *p<0.05. (B) T cells isolated from stage IV PDAC patient c76 were transduced to express HER2 CAR, and representative flow cytometry analyses are shown (upper). Effector T cells from patient c76 were overlaid either on c76-derived autologous PDAC targets or on allogeneic PDAC 215 targets. Target cell death of GFP-expressing PDAC targets was quantified by fluorescence microscopy (lower left) and WST-1 viability assay (lower right). Due to the limited number of T cells that could be isolated from this patient, replicates could not be performed in this instance. (C) LDH release assay performed on stage IV PDAC targets and control cells using switchable CAR-T effectors with different concentrations of the HER2 switch. (D) Cytotoxicity assays using switchable CAR-T effectors and c76 targets at a 20:1 ratio, with HER2 switches added at the top concentration of 10^4^ pM. Target cell death was assessed by light microscopy and WST-1 viability assay; n=3, p<0.05. CAR-T, chimeric antigen receptor T; PDAC, pancreatic ductal adenocarcinoma; SSC-A, side scatter-area.

We used both conventional CAR-T cells and switchable CAR-T cells that act via an antibody (Fab)-based ‘switch’ molecule that allows control over in vivo activity (figure 1A). To first assess susceptibility of primary PDAC cultures to HER2 CAR-T cell targeting, we generated second-generation HER2 CAR-T cells with the 4-1BB costimulatory domain from normal donors, which were found to have similar levels of CD4 and CD8 expression as untransduced T cells (online supplementary figure 2a). HER2 CAR-T cells were found to disrupt the monolayer of cultures PDAC 215, 354 and c76, and lysis of target cells by HER2 CAR-T cells was confirmed and quantified using a WST-1 viability assay (figure 2A). In parallel, we also observed lactat dehydrogenase (LDH) release by the target cells on incubation with HER2 CAR-T cells (online supplementary figure 2b).

In vitro targeting of primary PDAC cells in an autologous setting is more informative for clinical use; however, it is a challenge to acquire PDAC cells and effector T cells from the same patient. As patient c76 was still alive at the time of study, we had the unique opportunity to investigate autologous CAR-T cell effects and to explore whether the relatively modest levels of HER2 expression in these target cells might be sufficient for successful HER2 CAR-T cell therapy. We isolated T cells from this patient for use as effector cells. After two rounds of transduction, about 50% of T cells were found to express HER2 CAR (figure 2B). Applying patient c76-derived HER2 CAR-T cells to a confluent layer of both GFP-luciferase expressing c76 cells and PDAC 215 cells led to lysis of the monolayer, while untransduced T cells had no effect (figure 2B). Thus, our data suggest that patient-derived T cells can be effectively retargeted to autologous tumours.

To assess the activity of switchable CAR-T cells against PDAC cultures, T cells from healthy donors were transduced with a second-generation switchable CAR harbouring a 4-1BB costimulatory domain.^17^ In vitro cytotoxicity assays were carried out by coculture of switchable CAR-T cells with target PDAC cells in the presence of a dose titration of the HER2 switch with the PNE peptide fused to the C-terminus of the 4D5 HER2 Fab (HER2 switch) representing our optimal switch design from our previous publication.^16 17^ An inactive switch comprised of the wild-type (WT) HER2 Fab without the PNE was used as a control. The HER2 switch mediated potent, dose-dependent lysis of various PDAC cultures with maximum levels of cytotoxicity as assessed by LDH  release, correlating with relative HER2 expression levels (figure 2C). Comparable levels of cytotoxicity were observed on MDA MB 453 HER2 2+ cells. The WT HER2 Fab had no effect against PDAC cells, demonstrating the specificity of the switchable CAR-T cells+HER2 switch for HER2 expressing cells (figure 2D). We further demonstrated the specificity of the switchable CAR-T cells at high (10^4^ pM) concentrations of the HER2 switch against PDAC cells using fluorescence microscopy and WST-1 viability assays (online supplementary figure 2c). Switchable CAR-T cells+inactive switch had little activity against the PDAC cultures in these assays. Moreover, we demonstrated that switchable CAR-T cells + HER2 switch mediated significantly higher levels of LDH release from PDAC targets than switchable CAR-T cells + inactive switch  (online supplementary figure 2d).

### CSC-enriched 3D cultures retain susceptibility to HER2 CAR-T cell-induced cytotoxicity

**Figure 3:**
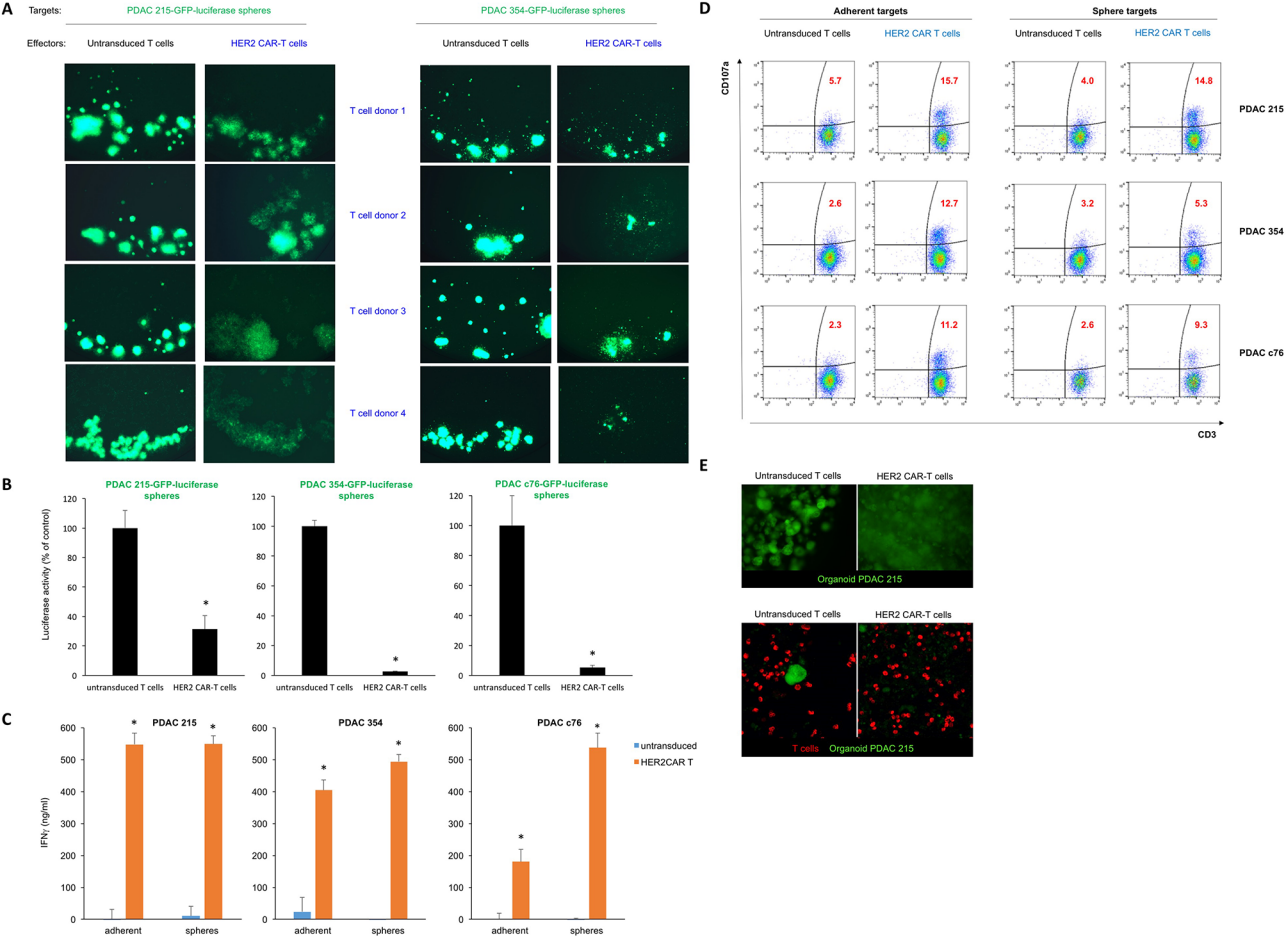
CAR-T cell-mediated cytotoxicity against CSC. (A) Lysis of spheres of GFP-expressing PDAC215 and PDAC354 target cell cultures in the presence of HER2 CAR-T cells, or untransduced T cells (control) at a 1:1 effector:target ratio. Effector cells from four different donors were used with consistent results. (B) Quantification of target cell viability by luciferase activity; n=3, *p<0.05 for spheres cocultured with HER2 CAR-T cells versus untransduced T cells. (C) Quantification of IFNγ secretion using ELISA during the coculture of effector T cells and PDAC 215, PDAC 354 and PDAC c76 adherent and sphere target cells; n=6, *p<0.05 for HER2 CAR-T cells versus untreated (UT) T cells. (D) CD107a degranulation assay following 6 hours of coculture of effector and target cells. (E) GFP-expressing PDAC 215 cells were embedded in Matrigel to allow PDAC organoid formation. After 7 days, labelled CAR-T cells or control T cells (red) were overlaid on top of the Matrigel and 3 days following coculture of T cells with organoids, fluorescence microscopy (top) and confocal microscopy (lower) was used to visualise the integrity of the organoids. CAR-T, chimeric antigen receptor T; CSC, cancer stem cell; PDAC, pancreatic ductal adenocarcinoma.

The dense matrix within CSC-enriched sphere cultures recapitulates features of the PDAC microenvironment and creates a physical barrier to T cell engagement with tumour cells. We therefore set up cocultures of PDAC spheres with effector CAR-T cells from four different T cell donors (1:1 effector:target (E:T) ratio) to test whether CAR-T cell killing of cancer (stem) cells is retained in a 3D environment. In all instances, HER2 CAR-T cells mediated lysis of both PDAC 215 and 354 sphere cultures, while untransduced T cells had no effect (figure 3A). We also assessed luciferase activity as a measure of target cell viability. We found a significant decrease in luciferase expression following coculture with HER2 CAR-T cells, demonstrating the ability of HER2 CAR-T cells to target CSC-enriched PDAC cultures (figure 3B). Prior to lysis, spheres were imaged by light microscopy and were observed to be targeted by HER2 CAR-T cells, consistent with the luciferase results (online supplementary figure 3a). Next, we explored the activation of the cytolytic killing machinery of CAR-T cells and found a strong upregulation of interferon gamma (IFNγ) for both adherent and sphere PDAC cocultures (figure 3C). Consistently, HER2 CAR-T cells specifically degranulate on engagement with HER2 antigen of adherent and sphere cultures. However, the proportion of T cells undergoing degranulation against sphere cultures was smaller than adherent cells, most likely due to the reduced antigen exposure in 3D cultures and potential immune evasion mechanisms of the contained CSC.^24^ Of note, higher levels of degranulation were observed on coculture with PDAC 215 cells that express higher levels of HER2 than PDAC 354, and untransduced T cells did not degranulate in either condition (figure 3D). To further illustrate the activation of CAR-T cells in the presence of PDAC sphere targets after 3 days of coculture, we performed multiplex staining for surface and intracellular T cell markers using Hyperion Imaging Mass Cytometry (IMC, Fluidigm), revealing strongly increased T cell proliferation combined with enhanced production of the cytotoxic molecule Granzyme B (online supplementary figure 3b).

To further probe CAR-T cell activity in the presence of a microenvironment that may serve as a physical barrier, we generated organoid cultures of PDAC 215 embedded within Matrigel, a basement membrane preparation rich in several extracellular matrix proteins. After 7 days of culture, PDAC 215 organoids were fully formed, and T cells were overlayed on top of the Matrigel. After 3 days, we observed lysis of the organoids in the presence of HER2 CAR-T cells but not for untransduced T cells (figure 3E).

### Conventional CARs and switch CARs targeting HER2 are equally efficacious in an orthotopic stage IV PDAC model

**Figure 4:**
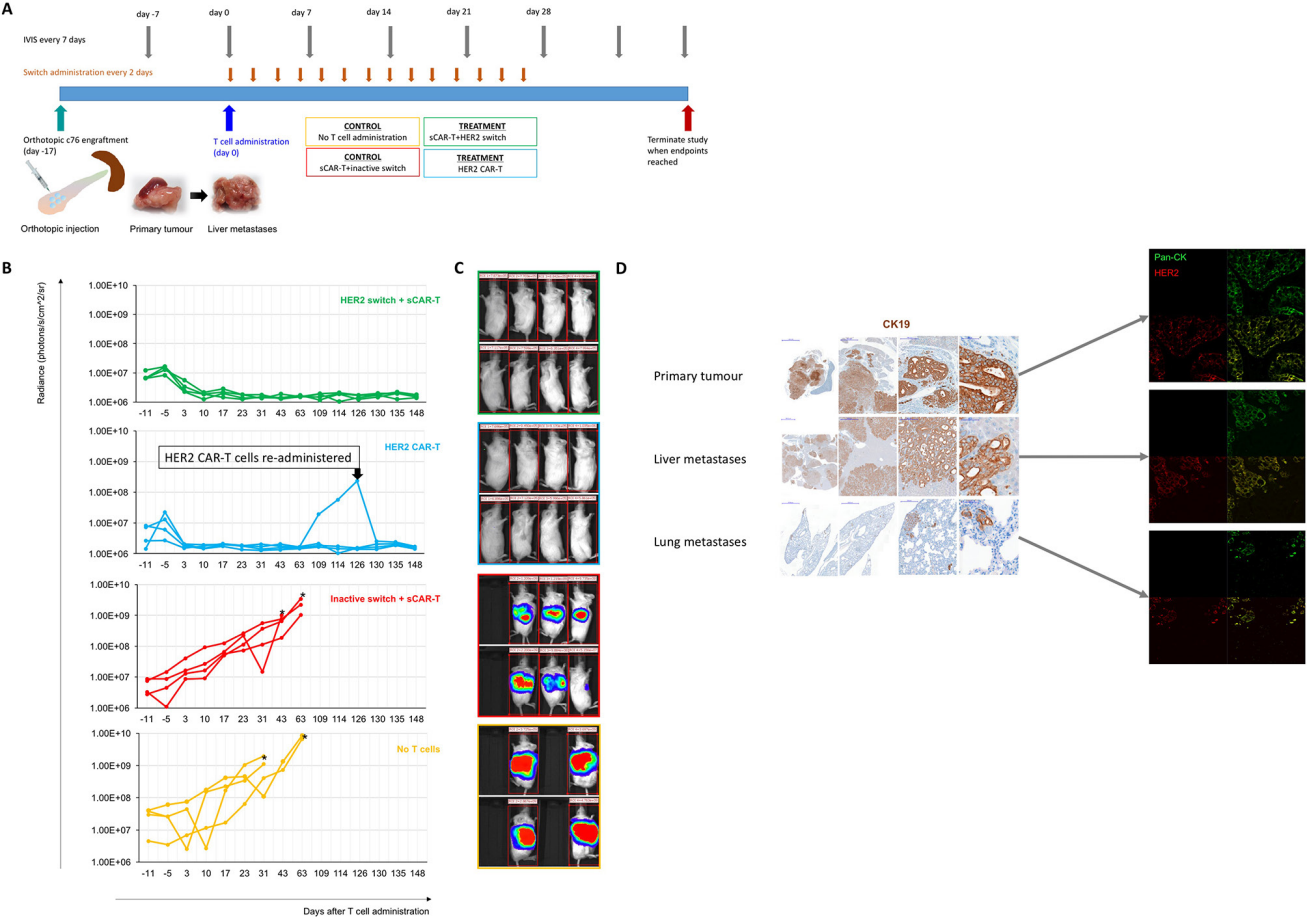
CAR-T cells efficiently target advanced PDAC in vivo. (A) Schematic of the isolation, culture, and orthotopic injection of PDAC cells derived from stage IV patients to locally expand in the pancreas and metastasize primarily to liver as well as lungs. c76 PDAC cells were administered orthotopically into the pancreas, followed by administration of CAR-T cells 17 days later. Switches were administered starting 4 hours after CAR-T cell administration and every 48 hours thereafter for a total of 14 doses. (B) Local tumour progression (left side of animal) and liver metastasis (right side of animal) were followed by measuring bioluminescence (IVIS) starting 11 days before and up to 148 days after CAR-T cell administration. Quantification of tumour progression in all cohorts up to 148 days; n=4, *p<0.05. (C) Representative images of the tumour bioluminescence are shown at day 63. (D) Primary orthotopic tumours, liver metastases and lung metastases were identified by immunohistochemistry for human cytokeratin-19 (CK19) and immunofluorescence for HER2 (red) and pan-CK (green). CAR-T, chimeric antigen receptor T; PDAC, pancreatic ductal adenocarcinoma.

To test the efficacy of HER2 CAR-T and switchable CAR-T cells in vivo, we used an orthotopic model of advanced PDAC. We chose to use stage IV c76 PDAC cells for two reasons: (1) c76 cells express HER2 at relatively modest levels, thus setting up stringent conditions on CAR-T therapy, and (2) c76 cells establish extremely aggressive primary tumours with rapid-onset metastasis to liver and lung reminiscent of late-stage PDAC in patients. We implanted 1e5 c76 PDAC cells into the pancreas of immunocompromised NSG mice to develop aggressive orthotopic tumours, which predominantly metastasised to liver and lung (figure 4A). Disease burden was monitored by non-invasive imaging of bioluminescence (IVIS). At day 17, groups were treated with HER2 CAR-T cells, switchable CAR-T cells, or no treatment. Animals receiving switchable CAR-T cells were dosed with either (1) the HER2 switch or (2) the inactive switch. Switch injections were initiated 4 hours after T cell administration and followed every other day thereafter for a total of 14 injections (figure 4A).

By day 3 following T cell administration, tumours in the switchable CAR-T+HER2 switch and conventional HER2 CAR-T cohort were diminished, and by day 10, the tumours had disappeared as assessed by IVIS. The mice continued to be healthy and tumour free for the duration of the experiment. By contrast, tumours in the ‘inactive switch’ and ‘no T cells’ cohorts continued to progress, with all animals developing large, rapidly growing orthotopic tumours, demonstrating the switchable CAR-T cell requires the switch for activity (figure 4B,C, online supplementary figure 4a). Consistent findings were obtained by high-resolution ultrasound (online supplementary figure 4b). At later time points, prominent liver metastases and less abundant lung metastases were also observed in these control mice, and HER2 expression was found to be maintained in primary tumour as well as metastases (figure 4D).

We observed tumour recurrence at the orthotopic site on a single animal in the HER2 CAR-T group on day 109, and rapid growth of HER2-expressing tumour cells was observed over the subsequent 17 days (online supplementary figure 4a). Readministration of HER2 CAR-T cells from the same donor on day 126 led to rapid tumour regression within 3 days (online supplementary figure 4a). Tumour recurrence was not observed in any other animal in either the HER2 switch or conventional HER2 CAR-T cohorts, and all the animals continued to be healthy with no signs of morbidity over the duration of the study.

**Figure 5:**
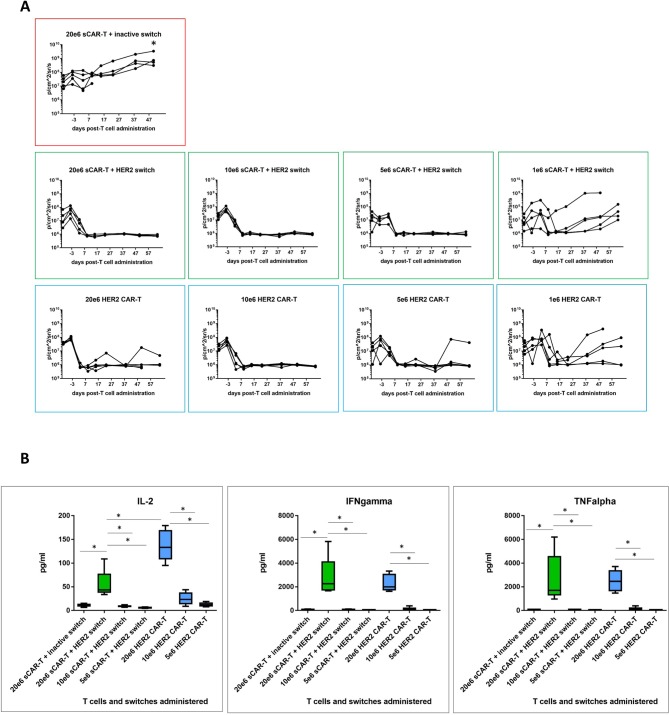
Comparative efficacy of decreasing doses of switchable CAR-T and HER2 CAR-T in vivo. (A) CAR-T cells were administered in decreasing doses to test comparative efficacy of both CAR-T cell effector types. Local tumour progression (left side of animal) and liver metastasis (right side of animal) were followed by IVIS as before. Quantification of tumour progression in all cohorts for up to 64 days; n=5, *p<0.05 for all treatment groups versus switchable CAR-T+inactive switch. (B) Human cytokine analysis (interleukin-2 (IL-2), interferon-gamma (IFNγ) and tumour necrosis factor-alpha (TNFα)) in murine peripheral blood plasma isolated 24 hours following CAR-T cell administration; n=5 per group, *p<0.05 for the indicated groups. CAR-T, chimeric antigen receptor T.

To compare efficacy of switchable CAR-T + Her2 switch and HER2 CAR-T cells, we next set up a dose titration of T cells (at doses of 20e6, 10e6, 5e6 and 1e6) administered to NSG mice bearing orthotopic PDAC c76 tumours as before. Animals administered switchable CAR-T cells received a total of 10 doses of HER2 switches. A single cohort of animals, used as a negative control, received 20e6 switchable CAR-T cells with inactive switches. Tumour regression was observed in all active treatment groups, with nearly complete clearance at 5e6 T cells per animal for both switchable CAR-T and HER2 CAR-T groups (figure 5A). Groups treated with 1e6 T cells exhibited variable tumours, which were comparable between switchable CAR-T and HER2 CAR-T groups. Two of five animals in the 20e6 (highest T cell dose) HER2 CAR-T cell cohort developed GvHD-like symptoms and were sacrificed at day 52 (online supplementary figure 5).

We next compared the in vivo activity of switchable CAR-T cells (dosed with 0.5 mg/kg HER2 switches) with HER2 CAR-T cells measured as cytokine release in the circulation 24 hours after CAR-T cell administration. Similar profiles of IFNγ and TNFα cytokine release were observed at each T cell dose, consistent with the similar antitumour activity mediated by either effector cell type, but IL-2 was higher in the HER2 CAR-T cell cohort compared with switchable CAR-T cells+HER2 switches. Importantly, we found little to no cytokines in switchable CAR-T cells treated with inactive switches in mice administered 20e6 switchable CAR-T cells, demonstrating the strict requirement of switches for switchable CAR-T cell activation (figure 5B).

To assess long-term CAR-T cell activity in vivo, we harvested peripheral blood 21 days after the final switch administration in the highest (20e6) dose cohorts (online supplementary figure 6). Drawing 100 µL blood from three mice per group followed by sorting for human CD2, we detected human CAR-T cells above a threshold of 100 events only in one animal in each group. This might be due to technical limitations (low cell numbers for sorting) and donor-dependent variability, but the samples available for analysis indicated a 5.7-fold increase in the number of switchable CAR-T cells in the presence of HER2 switches (online supplementary figure 6a, b). Multiplex phenotypic analysis by Hyperion Imaging Mass Cytometry indicated that T cells in both switchable CAR-T+HER2 switch and HER2 CAR-T cohorts were dominantly CD8^+^, consistent with the 4-1BB costimulatory domain driving expansion of CD8 cells^25^ (online supplementary figure 6b). Phenotypically, both groups were CD45RA^−^CCR7^−^, indicative of effector memory cells. Here, we observed striking differences for the two groups in the expression of the intracellular cytolytic enzyme Granzyme B and the proliferation marker Ki67. Our data suggest persisting activation and replication at high levels in HER2 CAR-T cells, while switchable CAR-T cells, 21 days after the last administration of the HER2 switch, persisted but displayed a resting phenotype (online supplementary figure 6b,c). Persistence of proliferation and production of cytotoxic molecules in HER2 CAR-T cells might be due to the graft versus host disease (GvHD)-induced activation. Alternatively, it could be explained by the presence of residual dormant cancer cells that can still be detected in disease-free long-term cancer survivors.^26 27^ However, persisting signalling in CAR-T cells with shared normal tissue targets might have detrimental effects in the clinic. Therefore, our data show that unlike conventional CAR-T cells, the in vivo activity of switchable CAR-T cells is contingent on adminstration of switches.

### Targeting HER2-expressing PDAC tumours in an aggressive metastasis model

**Figure 6:**
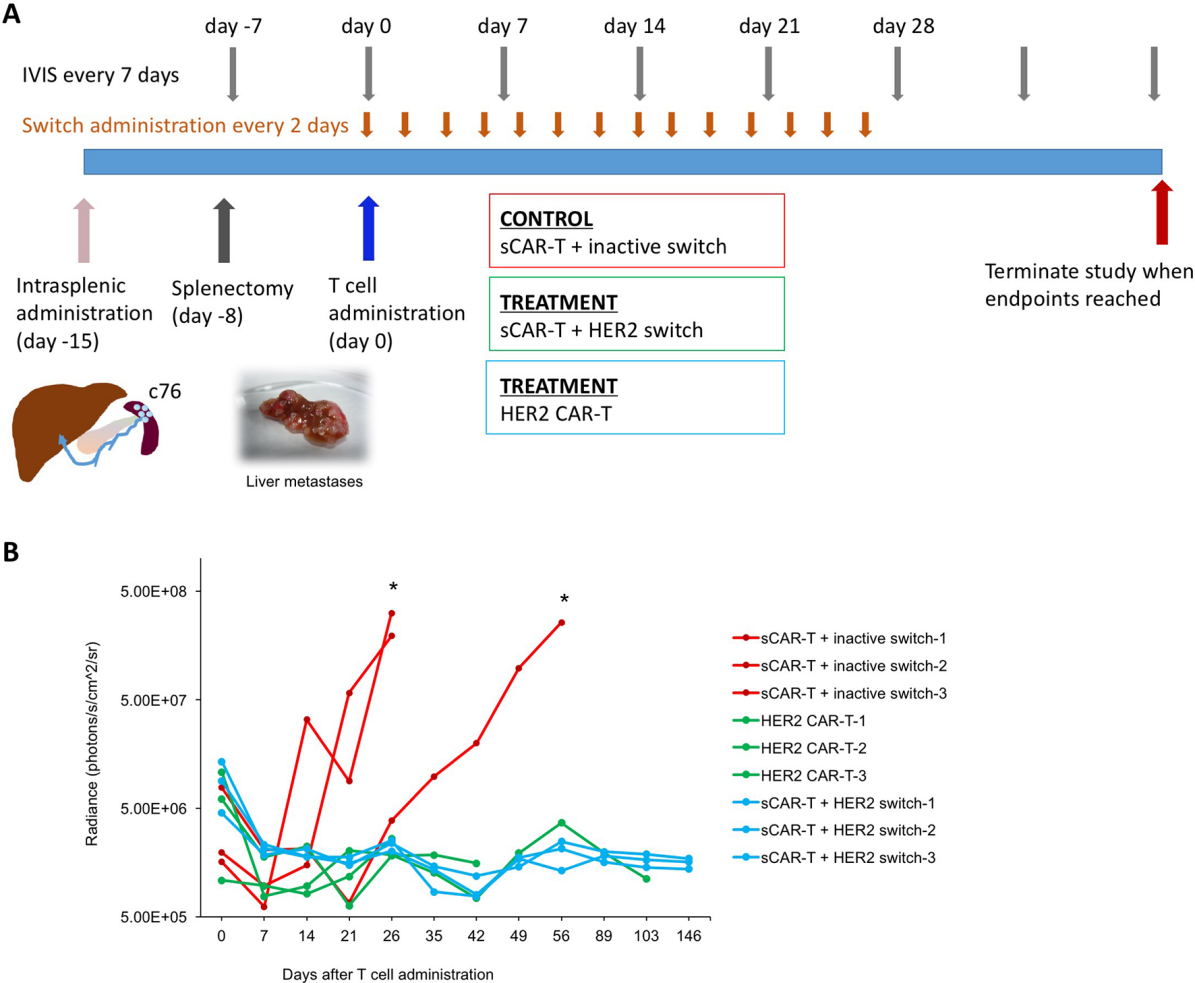
CAR-T cells efficiently target PDAC metastasis. (A) Schematic illustration of experimental setup and time-course. Stage IV c76 PDAC cells were injected into the spleen (day −15). Splenectomy was performed 7 days later (day −8), followed by CAR-T cells 15 days later (day 0). Switches were administered starting 4 hours after CAR-T cell administration, and every 48 hours thereafter for a total of 14 doses. (B) Quantification of tumour progression in all cohorts; n=3, *p<0.05. CAR-T, chimeric antigen receptor T; PDAC, pancreatic ductal adenocarcinoma.

Finally, we investigated the efficacy of targeting HER2 in a most challenging metastasis model, where c76 PDAC cells derived from Stage IV patients were administered intrasplenically to NSG mice and rapidly form metastases due to direct drainage from the spleen into the portal vein. After 7 days, splenectomy is performed to allow large liver metastases to form, which typically leads to end-stage disease at about 5 weeks (figure 6). Tumour development was monitored in the mice by IVIS.

Fifteen days after tumour administration, CAR-T cells were administered, followed by injection of HER2 and inactive switches 4 hours later and continued as injections every other day for the subsequent 14 days. Rapid tumour clearance was observed in the switchable CAR-T cells+HER2 switch and conventional HER2 CAR-T cell cohorts. By contrast, two out of the three animals in the switchable CAR-T cells+inactive switch cohort rapidly developed liver metastases as well as local tumours at the site of splenectomy (figure 6). The mice were sacrificed at day 26, confirming the presence of liver metastases (online supplementary figure 7). Cytokeratin-19 (CK19) immunohistochemistry (IHC)^28^ using an antibody specific to human CK19 further validated the presence of micro- and macrometastases in the liver. The remaining animal in the switchable CAR-T+inactive switch cohort developed large liver metastases by day 64. All the animals in the switchable CAR-T+HER2 switch and conventional HER2 CAR-T cohorts remained tumour free for the duration of the study, which was defined as 5 months following T cell administration. The animals in the conventional HER2 CAR-T cohort were sacrificed at 30, 43 and 103 days post-T cell administration due to the onset of GvHD-like symptoms. The absence of tumour was confirmed by IVIS, gross dissection and histology with CK19 IHC. Our data therefore confirm the efficacy of HER2 targeting in disseminated metastatic PDAC tumours via either conventional CAR-T cells or the switchable CAR-T systems.

## Discussion

CAR-T cells face unique challenges in the treatment of pancreatic cancer. To ensure favourable clinical outcomes, off-tumour effects inherent to all CAR-T cells have to be minimised, and homing and penetration into the desmoplastic stroma have to be successful. Recapitulation of the most aggressive features of human disease in mouse xenograft models is a useful tool for development of new therapies, as well as for individualised therapy for the specific patient. Work in our lab and others has shown that patient-derived xenograft models can reproduce many characteristics of human disease such as histological features, invasive tumour growth and, in some instances, metastases, although the caveat being that the cellular components of the TME cannot be modelled in immunodeficient animals. Intriguingly, many of our patients with PDAC are still alive when using their tissue for in vivo studies such as the present, thus the mice could serve as true Avatar models, in which the response to treatment is tested by mouse proxies.^29^ PDAC c76 that was used in the present study and was derived from a stage IV PDAC patient with extensive metastatic disease revealed that conventional and switchable CAR-T cells had similar efficacy in mediating tumour regression. A major advantage of switchable CAR-T was their specific activation in the presence of a switch, which represent favourable features for tumour antigens with some expression on healthy tissue.

Previous studies in our lab have shown that CSC are a distinct population within bulk PDAC tumours with distinct properties from differentiated PDAC cells^4 5 30^ and that CSC drive most resistance to conventional therapy.^31 32^ We have shown that primary PDAC cultures resemble PDAC tumours much more than cell lines, in that they have a relatively large CSC compartment, are highly tumourigenic in animal models and mimic the natural course of the disease.^3 33 34^ We have also shown that targeting the pancreatic CSC compartment efficiently prevents disease relapse resulting in long-term survival.^35 36^ After validating that CSC-enriched sphere cultures express HER2, we demonstrate that these sphere cultures can be targeted efficiently by HER2 CAR-T cells.

Next, in two distinct in vivo models, we found that a single administration of switchable CAR-T cells was sufficient to induce durable remission, with tumours becoming undetectable after just five HER2 switch injections. In both models, we continued to administer switches for a total of 10-14 injections resulting in long-term remissions in all treated animals. Importantly, after stopping in vivo administration of the HER2 switches for 3 weeks, we observed in vivo persisting, but resting switchable CAR-T cells as evidenced by lack of proliferation and Granzyme B production. These data demonstrate efficient elimination of all cancer cells, including the most tumourigenic CSC, validating switchable CAR-T cells as powerful yet controllable anticancer tools.

Ongoing work in several labs is focused on identifying novel truly specific targets for CAR-T cell therapy in solid tumours. Some antigens have a fairly restricted expression in normal tissues, such as mesothelin (pleura and peritoneum)^37^ and carcinoembryonic antigen (CEA) (colon),^38^ while others such as CD24 are also expressed in the stem cell compartment of normal tissues.^39 40^ To date, antigens such as CD19 have not been identified for solid tumours and may never be. Thus, several strategies have been developed to mitigate off-tumour on-target off-tumour toxicity for antigens upregulated in tumours but shared with normal tissue. These approaches include split CAR systems activated only in the presence of two tumour antigens^41^ and iCARs inhibited by antigens expressed on normal tissue.^42^ Consistent with previous reports,^15–17^ we found similar antitumour effects with switches and conventional CARs suggesting that the anti-tumour efficacy of the switchable CAR-T system was not compromised. Importantly, this demonstrates that the Fab-based switch is able to colocalise with switchable CAR-T cells to afford elimination of fibrotic pancreatic cancer lesions and cancer (stem) cells.

It is important to note that local and disseminated disease were represented in both our models; all the control animals administered orthotopic tumours developed extensive liver metastases, and two out of three control animals in the metastatic model developed local tumours due to rapid invasion of the tissues surrounding the spleen. Thus, our data highlight the versatility and efficacy of the switchable CAR-T system against aggressive stage IV PDAC. Adoptively transferred T cells have been shown to traffic to both antigen positive and antigen negative tumours as well as to a variety of peripheral tissues,^43^ which may explain the successful homing of the switch and switchable CAR-T cells and rapid regression of the disease on HER2- switch administration.

In summary, we demonstrate that switchable CAR-T systems and advanced mouse xenograft models that effectively recapitulate most features of advanced human PDAC are effective tools to enable development of potentially safer and more effective therapies for this deadly malignancy.

## Materials and methods

### Primary human cancer cells

Blood and tumours from patients with PDAC were obtained with written consent from all patients. The collection was performed under the Barts Pancreas Tissue Bank Protocol (REC reference 13/SC/0592, IRAS project ID 142061 - Samples c75, c76, c102, c135, c139, c142),  the Biobank of the Spanish National Cancer Research Centre (CNIO), Madrid, Spain (reference1204090835CHMH - Samples Panc 215 and 354), the ARC-NET Biobank at the ’Rossi’ University of Verona Hospital, Italy (reference 6.B.04 - Samples Panc 10953, 12560, 12556, 12707, and 12975 – reference 6.B.04), and the biobank at the Department of Surgery, Klinikum Rechts der Isar, Technical University Munich, Germany (Ethics Committee of the Faculty of Medicine of the Technical University of Munich, reference 1926/07 – Sample Panc 140114).

### Enzyme-linked immunosorbent assay

The IFNγ ELISA was performed on cell-free supernatants from cytotoxicity cocultures at indicated time points using a kit from eBioscience (88-7316-88) according to the manufacturer’s instructions. In addition, we performed  LEGENDplex multiplex immunoassays (Biolegend) to measure the levels of IFNgamma, TNFalpha, and IL-2.

### HER2 targeting sequences

The HER2 scFv protein sequence is:

ARPDIQMTQSPSSLSASVGDRVTITCRASQDVNTAVAWYQQKPGKAPKLLI

YSASFLYSGVPSRFSGSRSGTDFTLTISSLQPEDFATYYCQQHYTTPPTFGQGTKVEIKR

TGGGGSGGGGSGGGGSEVQLVESGGGLVQPGGSLRLSCAASGFNIKDTYIHWVRQAPGKG

LEWVARIYPTNGYTRYADSVKGRFTISADTSKNTAYLQMNSLRAEDTAVYYCSRWGGDGF

YAMDYWGQGTLVTVSSAAA.

The full-length HER2 CAR (with signal peptide, scFv, CD8a hinge, CD137 and CD3zeta domains):

MALPVTALLLPLALLLHAARPDIQMTQSPSSLSASVGDRVTITCRASQDVNTAVAWYQQKPGKAPKLLIYSASFLYSGVPSRFSGSRSGTDFTLTISSLQPEDFATYYCQQHYTTPPTFGQGTKVEIKR

TGGGGSGGGGSGGGGSEVQLVESGGGLVQPGGSLRLSCAASGFNIKDTYIHWVRQAPGKG

LEWVARIYPTNGYTRYADSVKGRFTISADTSKNTAYLQMNSLRAEDTAVYYCSRWGGDGF

YAMDYWGQGTLVTVSSAAATTTPAPRPPTPAPTIASQPLSLRPEACRPAAGGAVHTRGLD

FACDIYIWAPLAGTCGVLLLSLVITLYCKRGRKKLLYIFKQPFMRPVQTTQEEDGCSCRF

PEEEEGGCELRVKFSRSADAPAYKQGQNQLYNELNLGRREEYDVLDKRRGRDPEMGGKPR

RKNPQEGLYNELQKDKMAEAYSEIGMKGERRRGKGHDGLYQGLSTATKDTYDALHMQALP

PR.

### Cytotoxicity assays

Cytotoxicity was determined using the LDH cytotoxicity assay kit II (RayBiotech; 68CX-LDH-S500) according to the manufacturer’s instructions. Cytotoxicity assays using anti-PNE CAR-T cells and different concentrations of PNE-tagged HER2 Fab switches were performed using the CytoTox 96 Non-Radioactive Cytotoxicity Assay (Promega) based on the release of LDH. We used, as target cells, different PDAC cultures and the following breast cancer cell lines as positive controls with defined HER2 status: HCC1954 (HER2 3+), MDA MB453 (HER2 2+), MDA MB435S (HER2 1+) and MDA MB468 (HER2 0). CAR-T cells were incubated with target cells at an effector: target cell ratio of 10:1, while maintaining a total cell concentration of 10^6^/mL. PNE-tagged switches were added and incubated at 37°C for 20–24 hours in complete Roswell Park Memorial Institute (RPMI) with 10% (vol/vol) heat-inactivated fetal calf serum (FCS). WST-1 viability assays were performed on adherent PDAC cultures following cytotoxicity with T cells and subsequent removal of T cells, using a kit from Roche(05015944001). Assays to detect in vitro activity of firefly luciferase were performed using a kit from Promega (E1910).

Organoid cultures of PDAC215 were generated on Matrigel-covered 96-well plates overlaid with 5000 PDAC215 cells embedded in 50% Matrigel and 50% media. The culture was further topped off with organoid media modified from,^44^ consisting of adDMEM, B27, penicillin/streptomycin, 2 mM Glutamax, 10 mM HEPES, 1.25 mM N-acetylcysteine, 10 µM Y-27632, 100 ng/mL Noggin, 500 ng/mL R-Spondin, 0.5 µM A83-01, 10 nM gastrin, 50 ng/mL EGF, 100 ng/mL FGF10, 5 ng/mL bFGF, 20 ng/mL Wnt-3a, 1 µM PGE2 and 1 mg/mL Primocin. Prior to coculture with PDAC organoids, T cells were stained with CellTracker Red CMTPX dye (C34552, Thermofisher Scientific) according to the manufacturer’s instructions.

### Flow cytometry

Sphere-derived pancreatic cancer cells and differentiated cancer cells were dissociated with TrypleE Express (Thermofisher), topped up with FBS-containing media and washed once with cold PBS. Spheres were filtered through 40 µM cell strainers. Herceptin Biosimilar (HER35-M, Source Bioscience Lifesciences) was used to detect HER2 expression, and cells were incubated for 1 hour at 4°C. The cells were washed again with cold PBS and incubated with Alexa Fluor 647-conjugated goat antihuman antibody (10337882, Fisher Scientific) used as a secondary antibody at a 1:200 dilution for 30 min at 4°C in the dark. For some experiments, costaining with CD133-PE (Miltenyi Biotec; 1:400) was performed for 30 min at 4°C in the dark. Following one more final wash with cold PBS, cells were resuspended in DAPI-containing FACS buffer and analysed by flow cytometry on a BD LSR Fortessa (BD Biosciences).

Degranulation assays for expression of CD107a (Biolegend 328611) were performed following coculture of PDAC cells with CAR-T cells or untransduced T cells for 24 hours. Briefly, 5 µL CD107a-Alexa Fluor 647 was added into 200 µL medium containing the cocultured PDAC and T effector cells. Thirty minutes later, Monensin (Biolegend) was added for another 90 min, followed by Brefeldin (1×; Biolegend) for 4 hours at 37°C. Eventually, we stained CD3+ T cells using CD3-PECy7 (Biolegend 317333). For all surface staining, antibodies were used at 1:50 in PBS for 10 min at room temperature and live cells negative for 4′,6-Diamidino-2-phenylindol (DAPI; 1 µg/mL, Sigma) were analysed on the Fortessa or sorted with the ARIA II cell sorter (both BD). To combine surface expression of HER2 with quantitative high resolution morphology, we used imaging flow cytometry (Imagestream, Amnis) and used Cytokeratin-FITC (Miltenyi Biotec130-112-931; 1:10) to identify epithelial cells following fixation with 4% paraformaldehyde for 15 min at RT and subsequent permeabilisation using PBS+0.1% Tween-20 (PBS-T).

### In vivo experiments

Firefly luciferase expressing PDAC cells were established by transducing cells with a PGK-GFP-IRES-Luciferase Lentivector system from Addgene. Cells were sorted for GFP expression with a FACS BD Aria II instrument (BD, Heidelberg, Germany) and subsequently expanded in vitro. Then, 50 µL of 1×10^5^ (orthotopic injection) or 0.25×10^5^ (splenic injection) PDAC-Luc cells, respectively, were surgically injected either into the spleen or pancreas of NSG mice aged 6–8 weeks (NOD.Cg-Prkdcscid Il2rgtm1Wjl/SzJ; Charles River). To perform surgery, the mice were anaesthetised with isofluorane (2%) and an incision was made into the left flank of the skin following sedation and analgesia. Then, a second incision through the peritoneum was carried out and the spleen with adjacent pancreas was visualised for the injection of PDAC cells. After closing the peritoneum with 6/0 sutures (B/Braun;0022002), the skin was closed by surgical staples. An IVIS-200 system (PerkinElmer, Waltham, Massachusetts, USA) was used for weekly in vivo luciferase imaging. Mice were anesthetised with isoflurane (2%) and injected intraperitoneally with 150 mg/kg of luciferin (Caliper Life Sciences) diluted 15 mg/mL in PBS. Sequential images were obtained after luciferin injection every 30 s (maximum light emission, ∼20 min after luciferin injection). Luciferase activity is in photons per second per square centimetre per steradian (p/s^−1^ cm^−2^ sr^−1^). Living Image software (Caliper Life Sciences) was used for image analysis. Once tumours become detectable, mice were randomised to various treatments. All animal procedures were conducted in accordance to the 3Rs and were approved by the Institute’s Institutional Animal Care and Use Committee (PPL70-8129).

### Imaging mass cytometry (IMC)

To purify human T cells from the mouse blood, we first sorted for CD2 expression by FACS, followed by IMC of the remaining CD2^+^ T cells according to the manufacturer’s instructions (Fluidigm) using the antibodies at 1:100. The following metal-conjugated antihuman antibodies were used (all from Fluidigm): CD45-Y89, CD3-Er170, CD8-Er168, CD4-Nd145, Granzyme-Yb171, Ki67-Dy162, PD1-Yb174, Tim3-Sm154, Lag3-Nd150, CD45RA-Eu153 and CCR7-Er167. Prior to the in vivo retrieval of human CAR-T cells from Stage IV patient-derived PDAC in vivo models following allocated treatment, we validated above antibodies on HER2 CAR-T cells in vitro in the presence or absence of activating human PDAC sphere cultures. Analysis was carried out using the MCD viewer software, and a heat map was generated by Morpheus visualisation and analysis software.

### Low-passage PDAC cultures

Pancreatic tumours were obtained from PDX as described previously.^3^ Tumours were homogenised using a GentleMACS Dissociator followed by enzymatic digestion with collagenase P for 15 min at 37°C and cultured in DMEM+10% FCS. Culture under low adhesion conditions to enrich for CSCs and human pancreatic cancer xenografts have been described previously.^22^ To culture spheres enriched in CSCs, cells were resuspended in 1× DMEM/F-12 (Gibco) supplemented with 20 ng/mL FGF-2 (CellGS), 0.4% amphotericin B, 1% penicillin/streptomycin, 2% B27 supplement (Gibco) and 200 mM of L-glutamine (Gibco). A cell suspension of 10 000 cells/mL was then prepared and distributed into ultra-low attachment surface flasks (Corning, New York, USA) for 1 week. Prior to use, spheres were filtered (40 µM).

### Generation of CAR-T cells

Briefly, we used the coding sequences for the anti-GCN4 scFv (clone 52SR4) and anti-HER2 scFv (clone 4D5) subcloned into the lentiviral plasmid pRRL-SIN-EF1α-WPRE and linked to the hinge, CD8 transmembrane region (TTTPAPRPPTPAPTIASQPLSLRPEACRPAAGGAVHTRGLDFACD) and the cytoplasmic regions of human 4-1BB and CD3ζ to create our second-generation CAR constructs (for sequence of HER2 CAR, see above). Lentiviral particles were produced via transfection of the lentiviral constructs into HEK293FT human embryonic kidney cells. Cells were transfected with pRRL-SIN-EF1α-WPRE, pMDL, pREV and pVSVG plasmids using FugeneHD (Promega). Human T cells from healthy volunteers were obtained by Ficoll-Pacque purification of PBMCs from normal donor whole blood from The Scripps Research Institute’s Normal Blood Donor Service at Scripps General Clinical Research Center, under the appropriate The Scripps Research Institute’s Institutional Review Board approval. PBMCs were further activated for 24 hours with CD3/CD28-coated magnetic beads (Life Technologies) before infection. Concentrated lentivirus was applied to the activated human T cells in the presence of 5 µg/mL protamine sulfate and 50 IU/mL IL-2 (R&D) and centrifuged at 1000× g for 2 hours at 32°C. The HER2 CAR expression level was determined by flow cytometry with Alexa Fluor 647 (Thermo Fisher)-labelled antihuman or antimouse IgG, while the switchable CAR expression levels were determined with Alexa Fluor 488-labelled GCN4.

### Production of HER2 switches

For GCN4 (PNE sequence: NYHLENEVARLKKL) fusions, gene fragments encoding 4D5 heavy or light chains, with or without GCN4, were subcloned into the pFuse vector. Fusions of the different combinations of heavy or light chains were expressed by transient transfection in the FreeStyle 293 Expression System. Briefly, HEK293F cells were transfected at a density of 10^6^ cells/mL with a 1:2 ratio plasmid DNA to 293fectin (Life Technologies). Cells were incubated at 37°C with 5% CO2 for 96 hours. The recombinant protein-containing supernatant was harvested at 48 hours and 96 hours and further purified by Protein G chromatography.

### Immunohistochemistry

Immunohistochemical staining on 2.5 µM sections was carried out using the Ventana Discovery XT system (Roche). Sections were deparaffinised, hydrated and loaded on the Discovery XT. Antigen retrieval was performed using citrate buffer solution. Endogenous peroxidases were quenched using H_2_O_2_ reagent (Ventana). Primary antibodies against cytokeratin 19 (Abcam ab9221; 1:1,000) were incubated for 20 min and detected using an antirabbit secondary antibody and the ChromaMap DAB detection kit (Ventana). Tissues were counterstained with haematoxylin, and image analysis was performed using Pannoramic Viewer Software (3DHISTECH).

For some experiments, after antigen retrieval, slides were manually stained with Herceptin Biosimilar (HER35-M, Source Bioscience Lifesciences) for 1 hour, followed by Alexa Fluor 647-conjugated goat antihuman antibody (10337882, Fisher Scientific) used at a 1:200 dilution for 30 min. Costaining with anti-human pan-cytokeratin-FITC antibody (Miltenyi Biotec130-112-931; 1:10) in PBS with 0.1 % Tween 20 was performed for 30 min. All experiments were carried out at room temperature. As soon as fluorescent antibodies were added, the slides were placed in the dark. Slides were mounted with ProLong Gold mounting media with DAPI (Thermo Fisher Scientific). Images were acquired using an LSM 710 confocal microscope (Zeiss) or a Pannoramic 250 flash III scanner (3DHISTECH).

### Statistical analysis

Unless stated otherwise, results are expressed as the means±SD. Statistical analyses were performed with SPSS V.22.0 comparing continuous variables by non-parametrical Mann-Whitney U and Kruskal-Wallis tests. The significance is given as p<0.05.
