# Supplementary material for: Switchable CAR-T cells mediate remission in metastatic pancreatic ductal adenocarcinoma
Source: Gut. 2018 Aug 18;68(6):1052–64. doi: 10.1136/gutjnl-2018-316595 (PMC6580747; doi:10.1136/gutjnl-2018-316595)
Supplement: Supplementary data [file gutjnl-2018-316595supp001.pdf]

Supplementary table 1

Core ID	PDAC grading	Scoring of HER2 #
M713	Well differentiated (grade 1)	HER2 3+
M762	Well differentiated (grade 1)	HER2 3+
M1077	Moderately differentiated (grade 2)	HER2 3+
M1084	Well differentiated (grade 1)	HER2 3+
M105	Mixture of carcinoma and non-neoplastic benign pancreas	HER2 3+ (adenocarcinoma), HER2 0 (benign ducts)
M109	Well differentiated (grade 1) and poorly differentiated (grade 3)	HER2 3+ / HER2 2+
M891	Moderately differentiated (grade 2)	HER2 2+
M1366	Moderately differentiated (grade 2) and well differentiated (grade 1)	HER2 2+ / HER2 1+
M1121	Well differentiated (grade 1) adenocarcinoma and non-neoplastic benign pancreas	HER2 2+ (adenocarcinoma), HER2 0 (benign ducts)
M704	Moderately differentiated (grade 2)	HER2 1+
M1143	Well differentiated (grade 1) adenocarcinoma and non-neoplastic benign pancreas	HER2 1+ (adenocarcinoma), HER2 0 (benign acini)
M592	Moderately differentiated (grade 2)	HER2 1+
M4	Poorly differentiated (grade 3)	HER2 1+
M193	Moderately differentiated (grade 2)	HER2 1+
M1406	Mainly non-neoplastic (benign), but also moderately differentiated (grade 2) adenocarcinoma, admixed some atypical glands, probably malignant.	HER2 0 (benign)<br>HER2 1+ (adenocarcinoma)
M907	Moderately differentiated (grade 2)	HER2 1+
Breast carcinoma	Grade 2-3 invasive ductal carcinoma of breast	HER2 3+

adapted from the algorithm for gastric adeno-carcinoma (IHC) from the College of American Pathologists

Supplementary table 1 – HER2 expression in PDAC. Tissue microarray containing clinical PDAC specimen cores (n=16) graded for HER2. Immunofluorescence (IF) staining for HER2 was compared to cytokeratin-19 (CK19) as a marker of epithelial tissues. Breast cancer tissue was added as positive control for high HER2 expression.

Supplementary figure 1

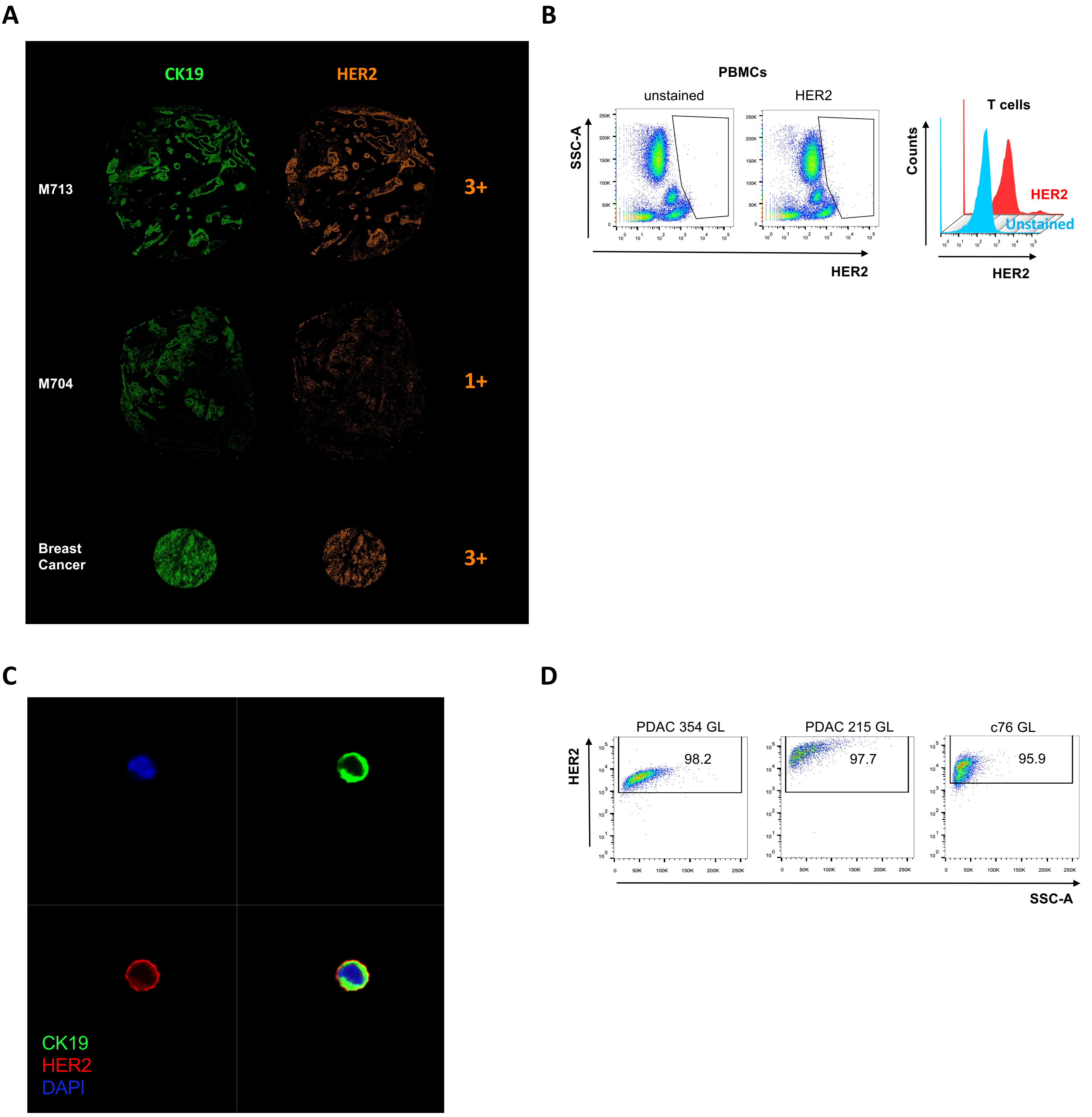

Supplementary figure 1 – HER2 expression in PDAC and blood cells. (A) Representative pictures of HER2 3+ and HER2 1+ (orange) clinical PDAC cores of our Tissue Micro Array (TMA). Cytokeratin-19 (CK19) was used as a marker of epithelial tissues (green). Breast cancer tissue (HER2 3+) is added as positive control for high HER2 expression. Images were captured using a Pannoramic 250 scanner. **(B)** Flow cytometry for HER2 expression in freshly isolated PBMC and cultured CD2-sorted T cells. **(C)** HER2 expression (red) on pan-cytokeratin^{+} (green) patient-derived circulating cancer cells (CTC). (**D**) HER2 expression on PDAC cultures 215, 354 and c76 was assessed after transduction with lentiviruses to express GFP and luciferase. Expression is given as percentage (%).

Supplementary figure 2

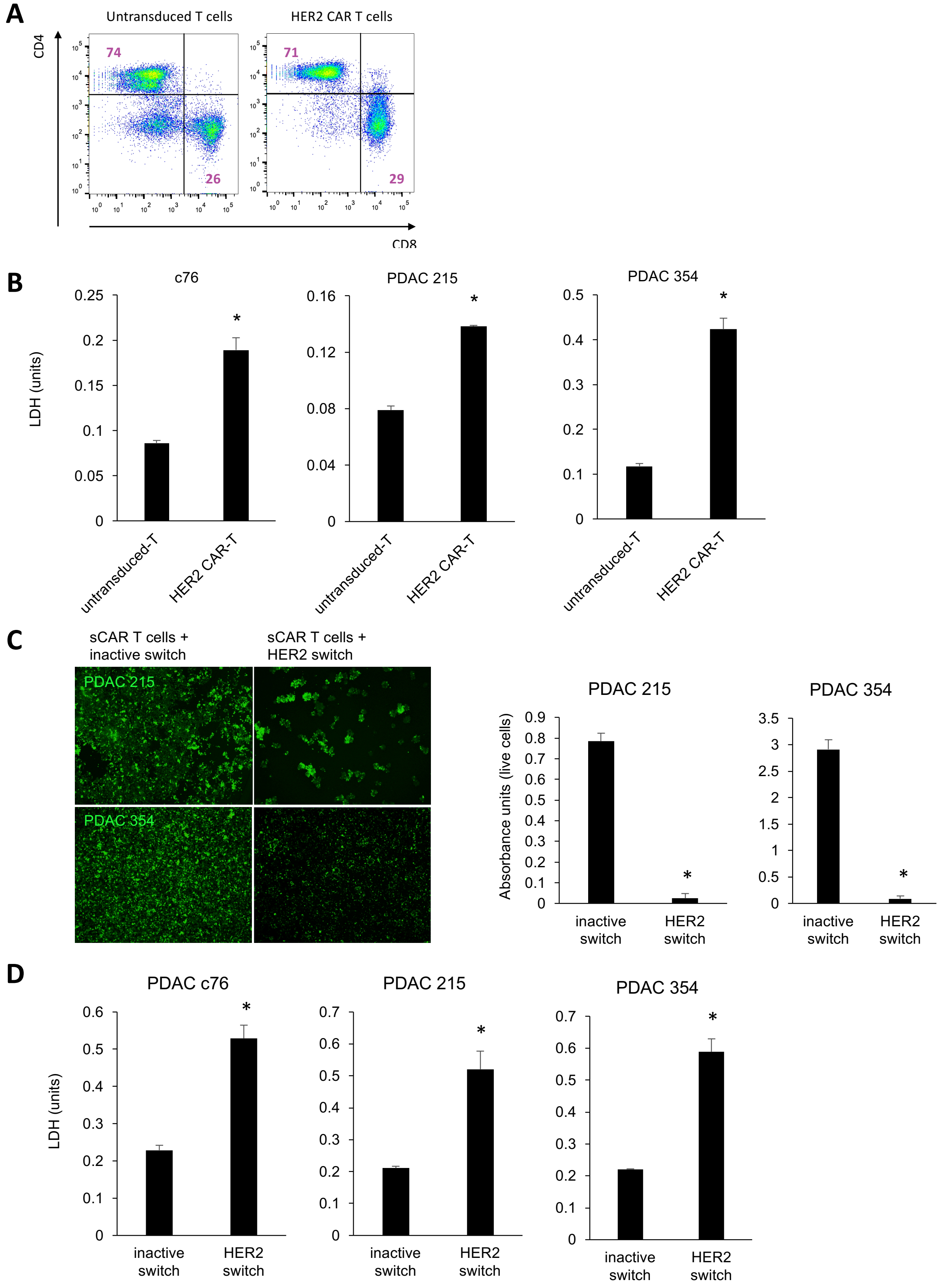

Supplementary figure 2 – CAR-T cell mediated cytotoxicity against PDAC cultures. (A) Distribution of CD4 and CD8 expression (CD4/CD8 ratio) on HER2 CAR-T cells vs. untransduced T cells. Levels of expression are indicated as percentage (insert). **(B)** Viability of co-cultures of PDAC 215, PDAC 354, and c76 cells with untransduced or HER2 CAR transduced T cells at an E:T ratio of 0.2:1 measured by LDH-WST (LDH cytotoxicity assay kit II); n=3, * p<0.05. LDH release in the supernatants was quantitated as the ratio of LDH content in the supernatants of the PDAC / T cell co-cultures versus the maximum possible release after lysis using a detergent-containing buffer. **(C)** sCAR-T cells were incubated with PDAC 215 and 354 targets at a 20:1 effector:target ratio, and either HER2 switch or inactive switch were added to the co-cultures at 10^4 μM. Target cell lysis was assessed by fluorescence microscopy and WST-1 viability assay at 48 hours of co-culture; n=3, * p<0.05. (**D**) sCAR-T-mediated target cell death was further assessed by LDH release; n=3, * p<0.05.

Supplementary figure 3

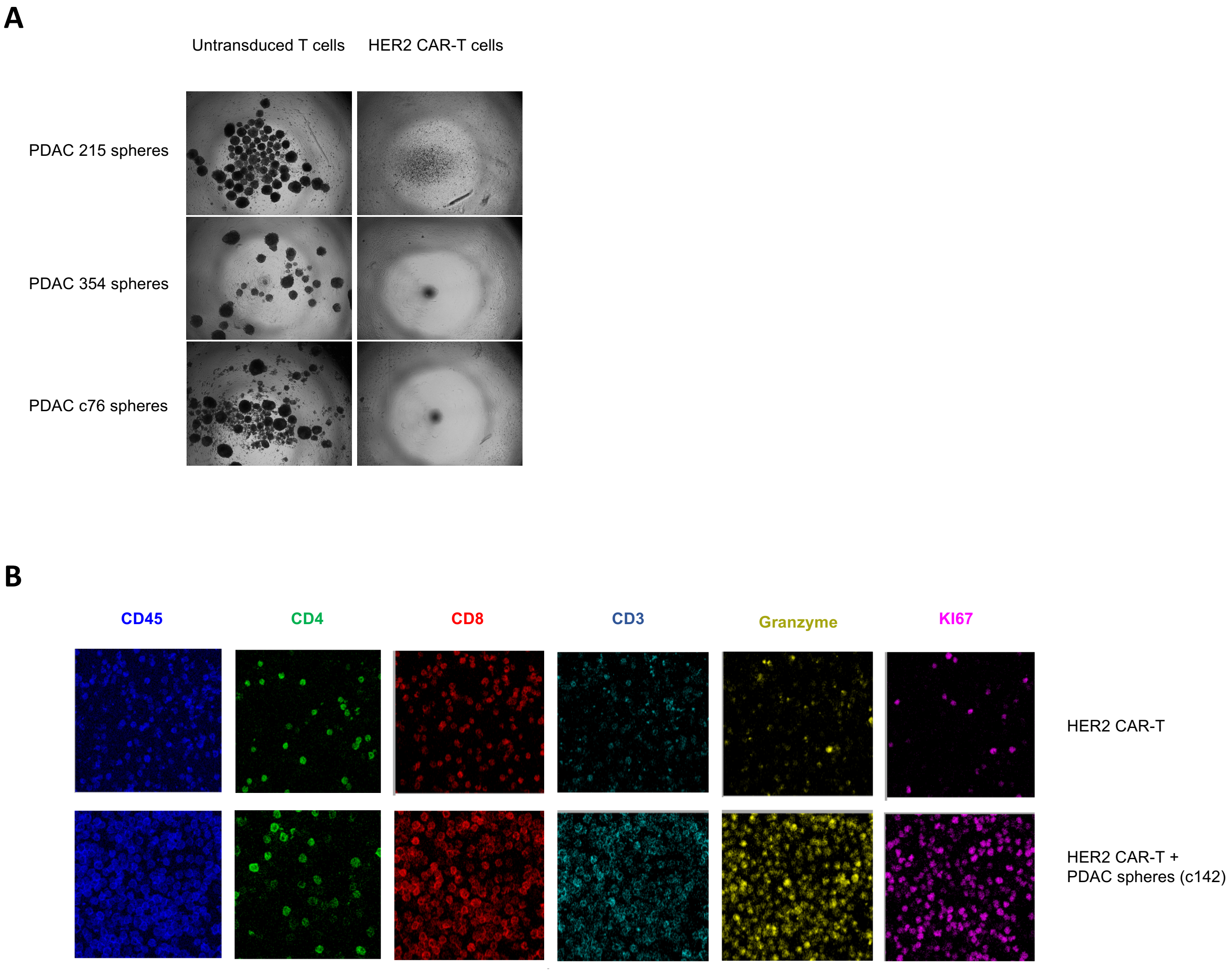

Supplementary figure 3 – CAR-T cell-mediated cytotoxicity against PDAC sphere cultures. (**A**) Sphere cultures were raised using PDAC c76, 215 and 354 cultures. After 7 days of sphere culture, effector T cells were added at a ratio of 1:1. After 48 hours of co-culture, spheres were washed with PBS and imaged by light microscopy. Reduction of sphere numbers indicates cytotoxicity. (**B**) HER2 CAR-T cells after 72 hours of co-culture with PDAC sphere targets **(lower panel)** or without **(upper panel).** Representative multiplex staining using Hyperion Imaging Mass Cytometry™. Increase in proliferation (KI67) and production of granzyme B indicates cytotoxicity.

Supplementary figure 4

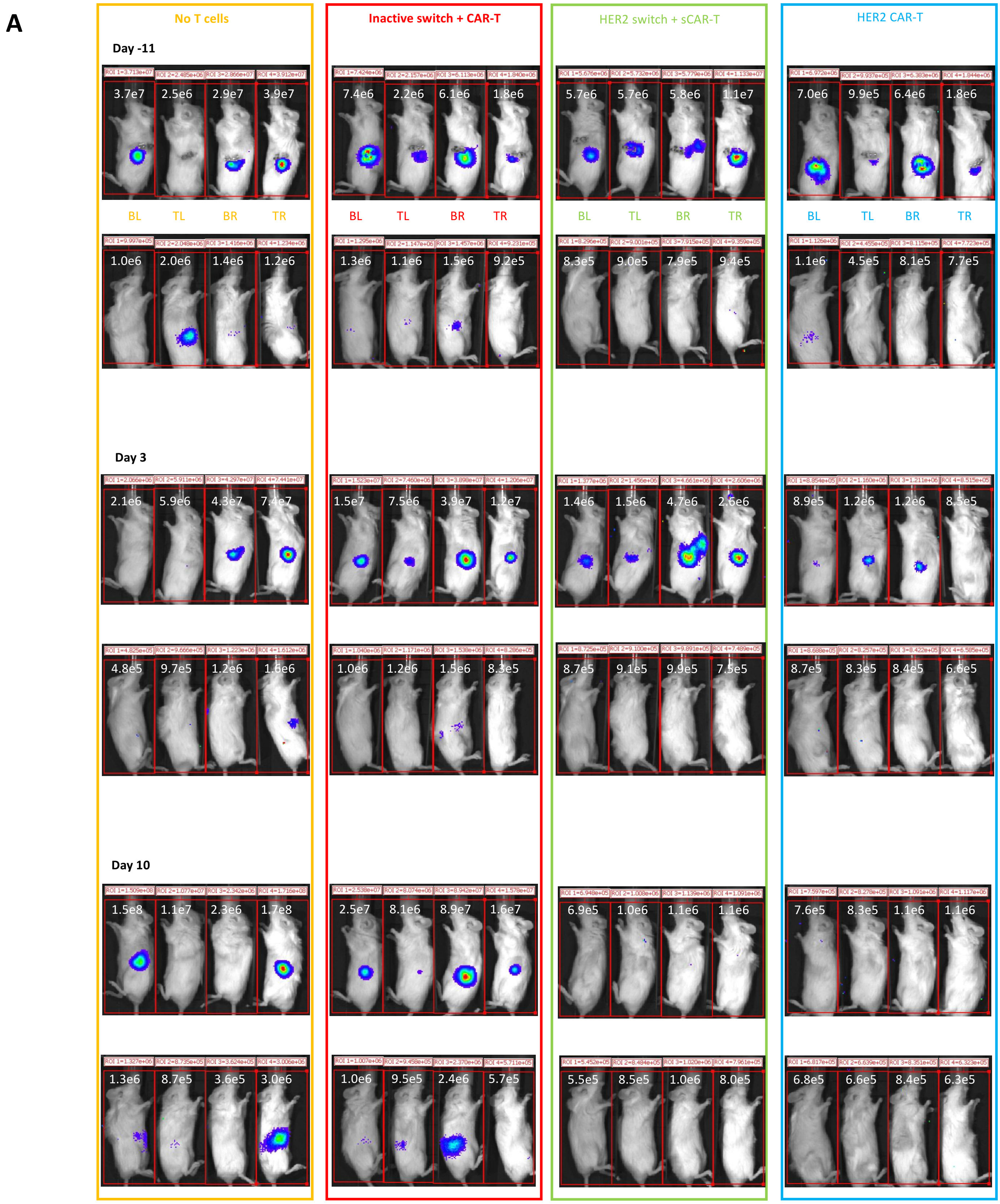

Supplementary figure 4. CAR-T cell treatment of orthotopic PDAC tumours.

(A) IVIS images of NSG animals engrafted with orthotopic luciferase^{+} PDAC c76 followed up for 148 days following treatment. Time course of bioluminescence measurements indicates tumour growth and metastasis of mice. **(B)** **(Top)** Correlation of tumour progression using IVIS and ultrasound-based determination of tumour volume; **(below)** representative ultrasound images. **(C)** HER2 expression in local tumours and metastases of relapsed mice; representative flow cytometric analyses are shown.

Supplementary figure 4 continued

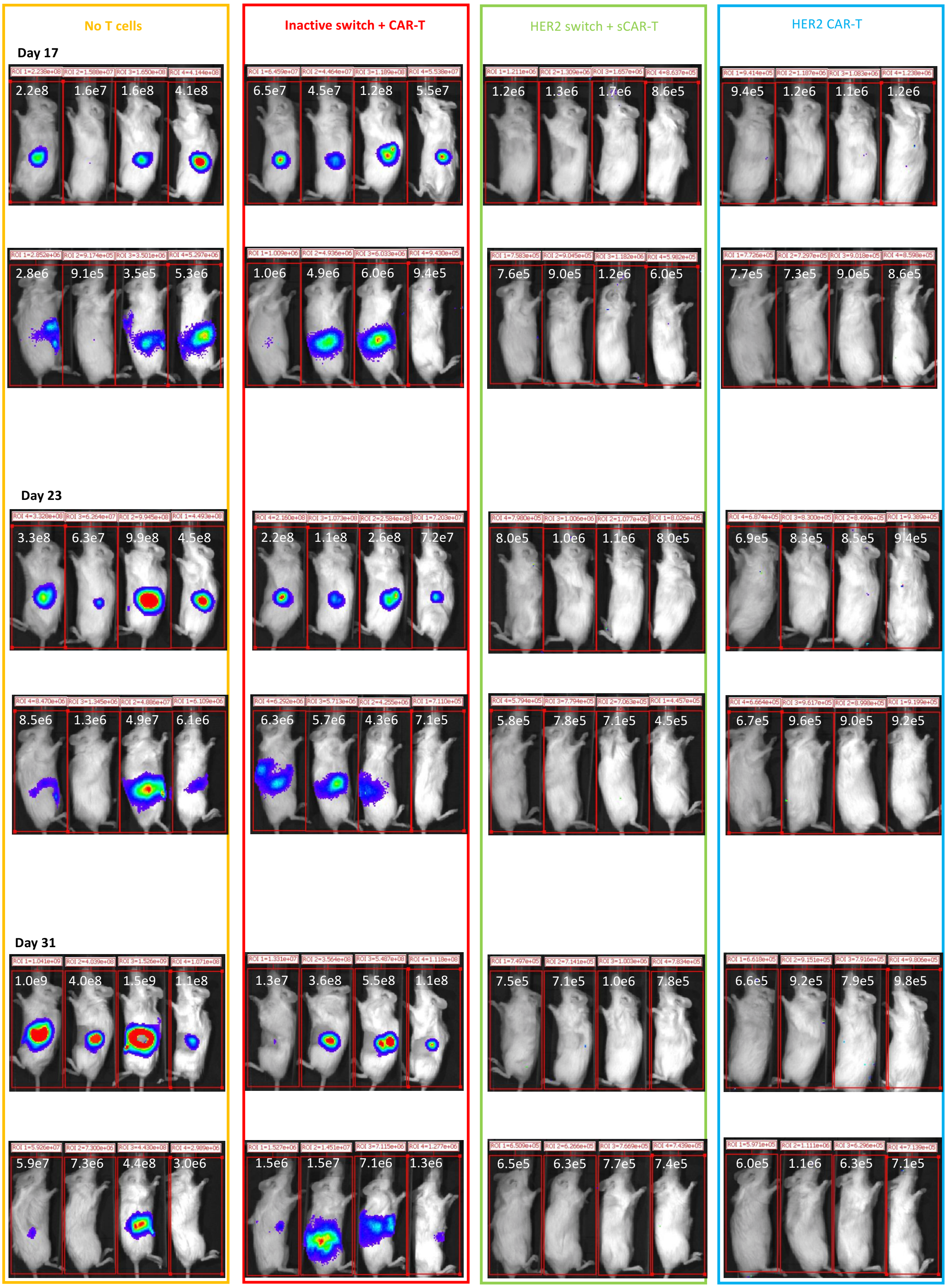

Supplementary figure 4 continued

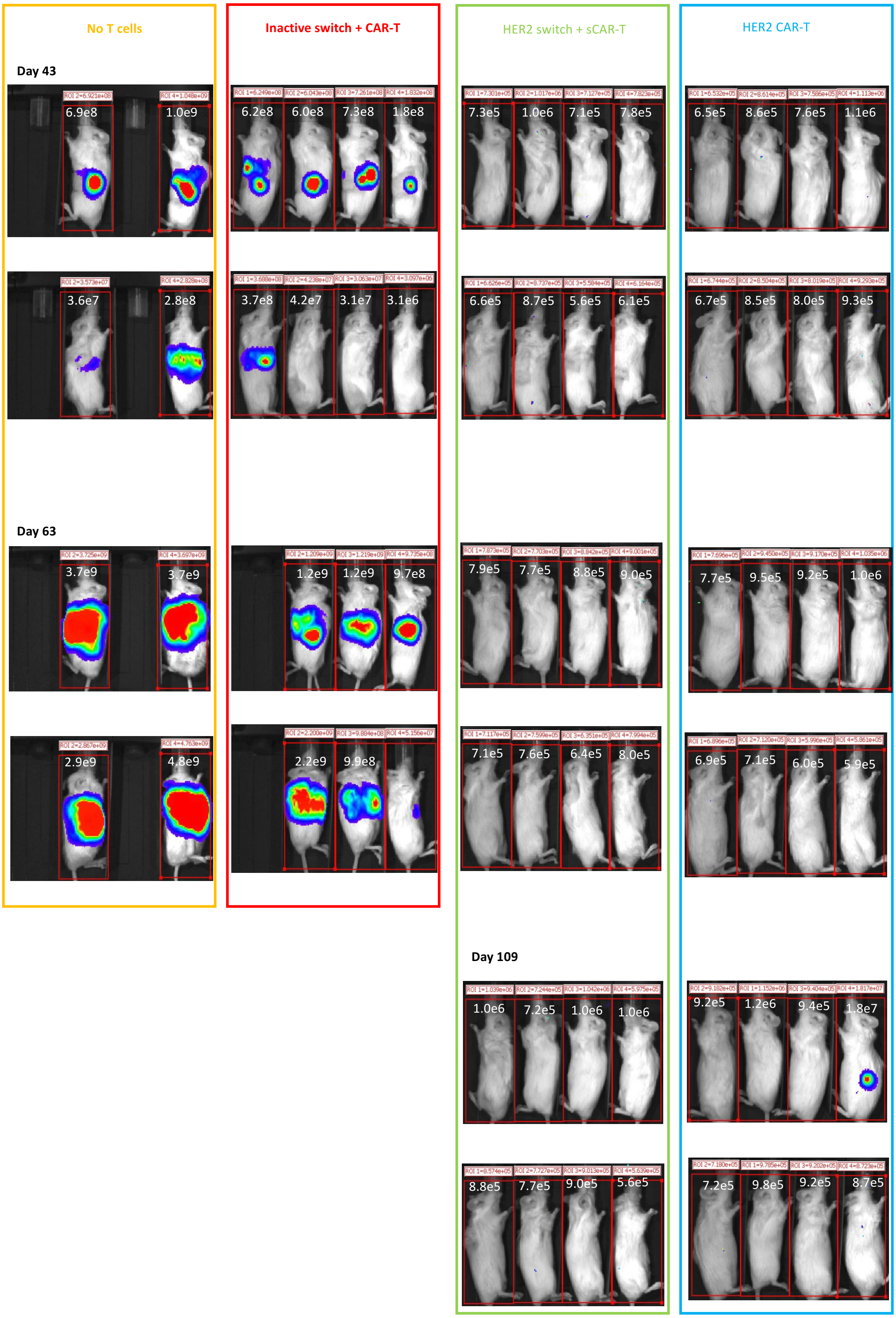

Supplementary figure 4 continued

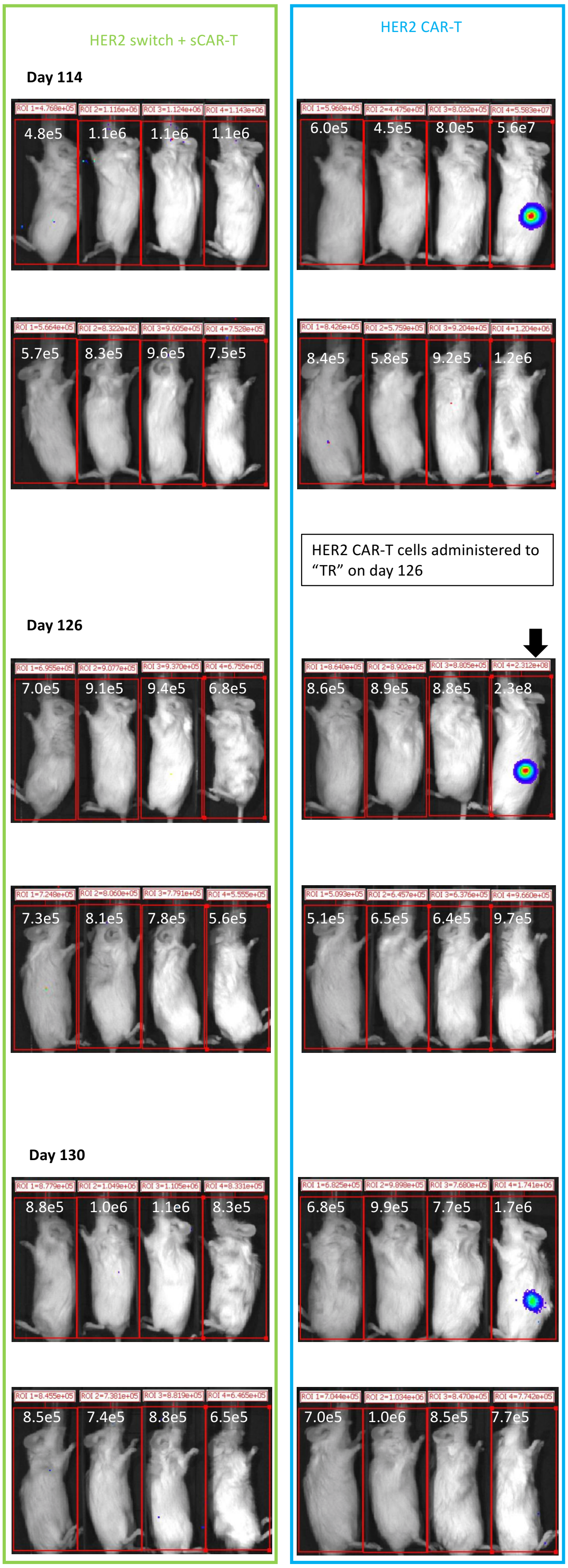

Supplementary figure 4

A (continued)

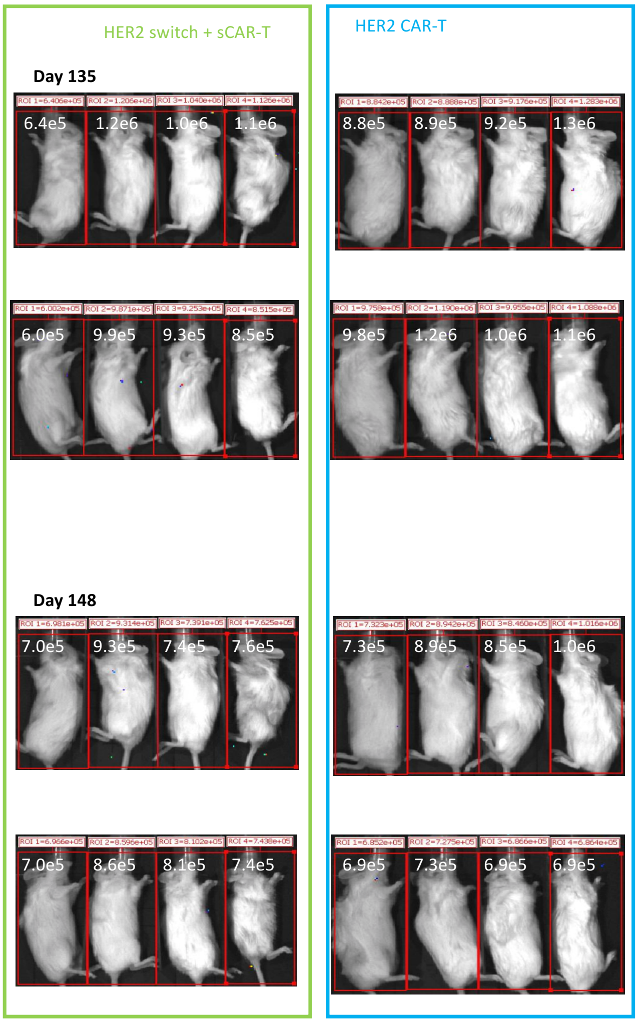

B

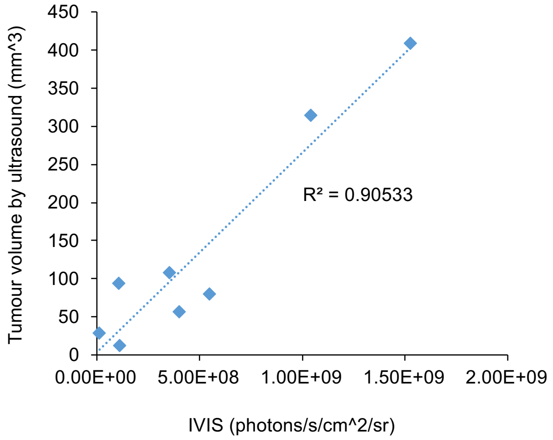

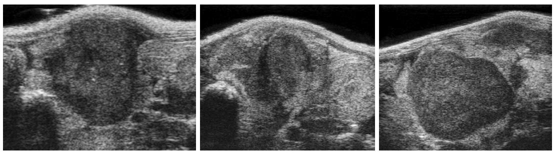

**Tumour volume by ultrasound (mm^3):**	**94.3**	**314.5**	**408.8**
**IVIS (photons/s/cm^2/sr):**	**1.07e8**	**1.04e9**	**1.53e9**

C

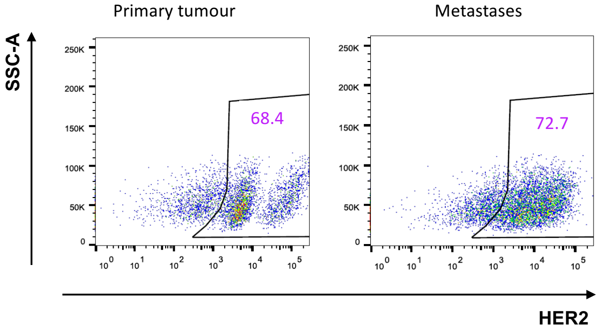

Supplementary figure 5

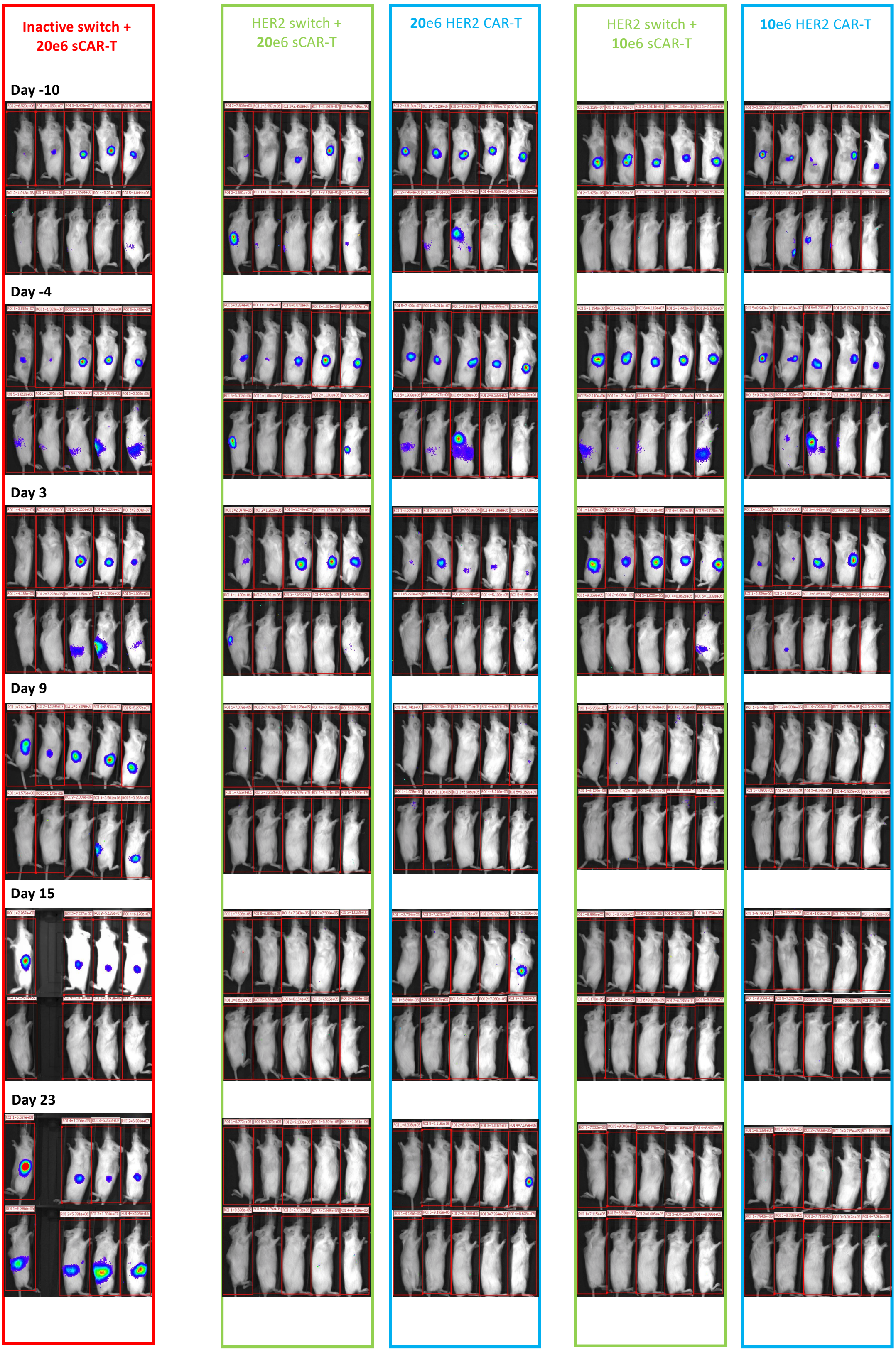

Supplementary figure 5. Follow-up of tumor progression after CAR-T treatment.

Tumor progression in different treatment cohorts after administration of graded doses of CAR-T cells is measured by assessing tumour bioluminescence by IVIS.

Supplementary figure 5 continued

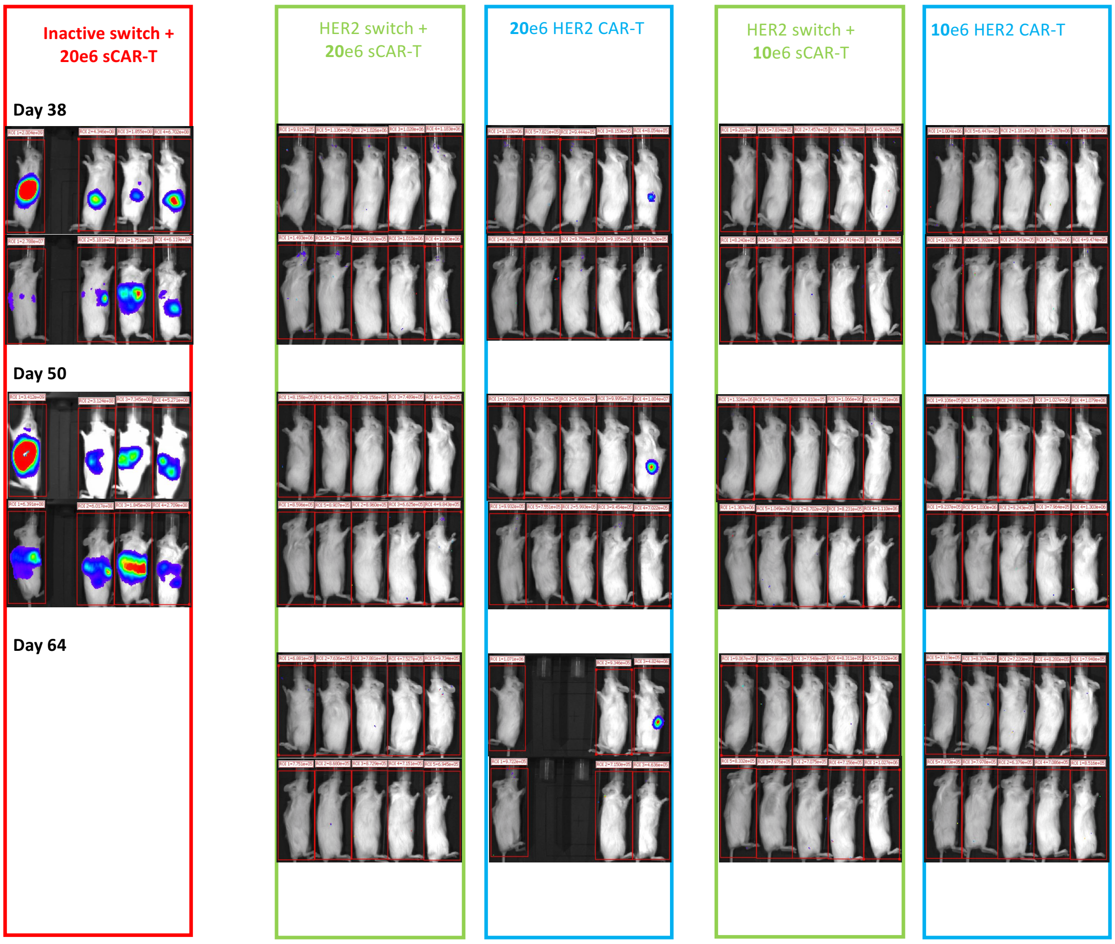

Supplementary figure 5 continued

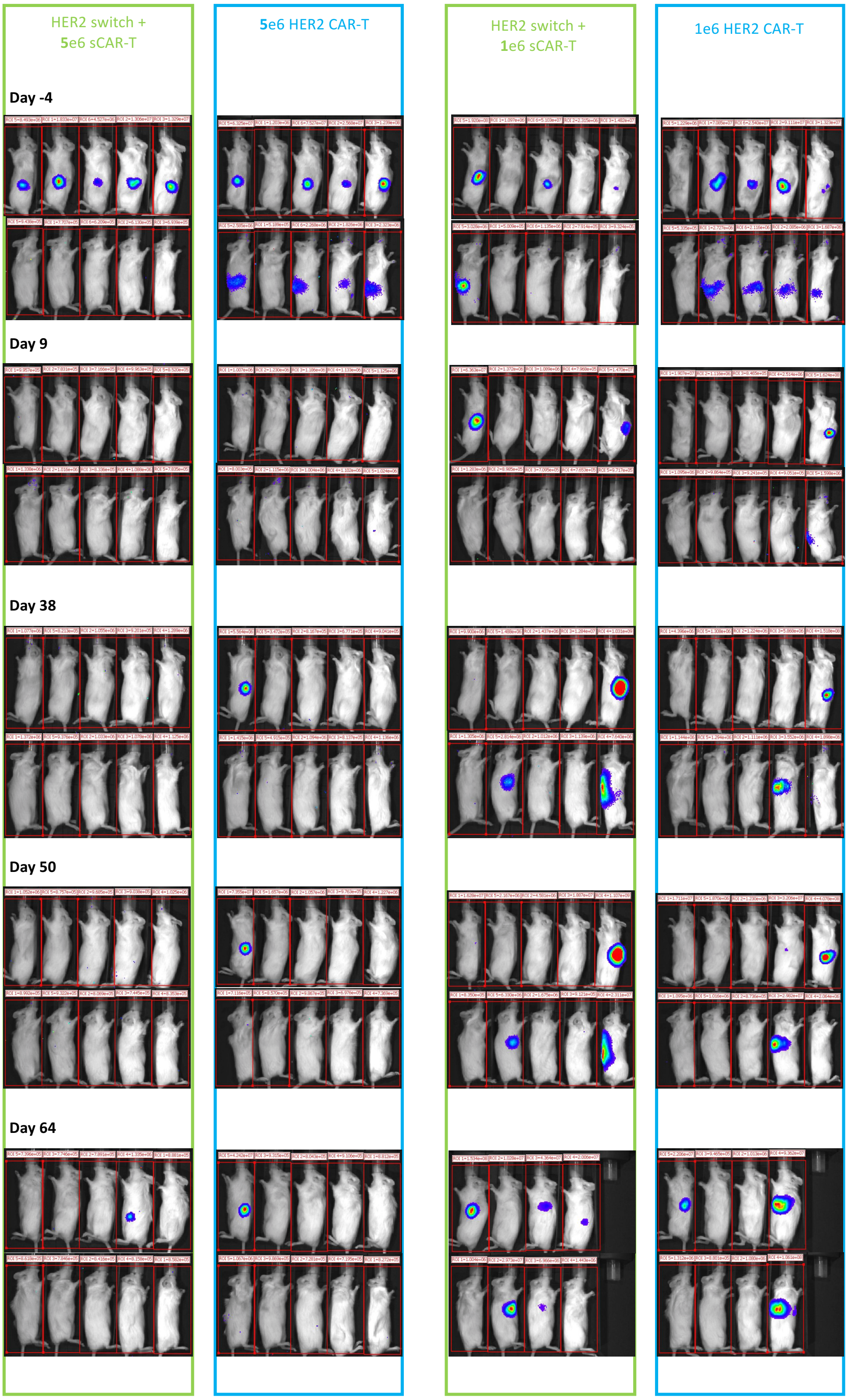

Supplementary figure 6

A

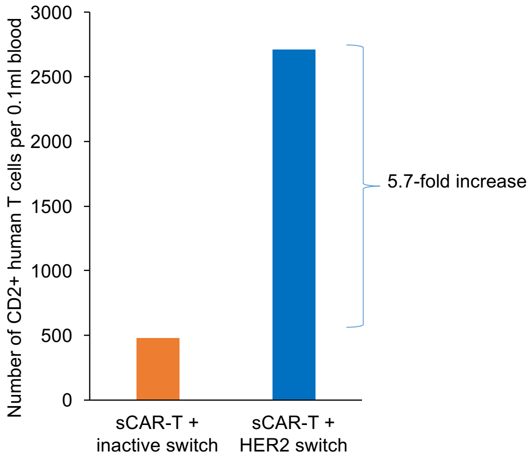

B

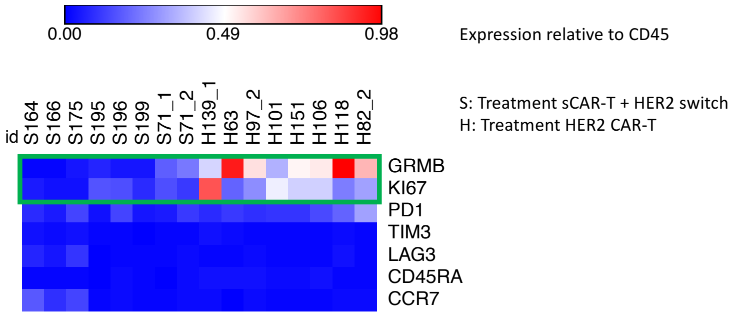

C

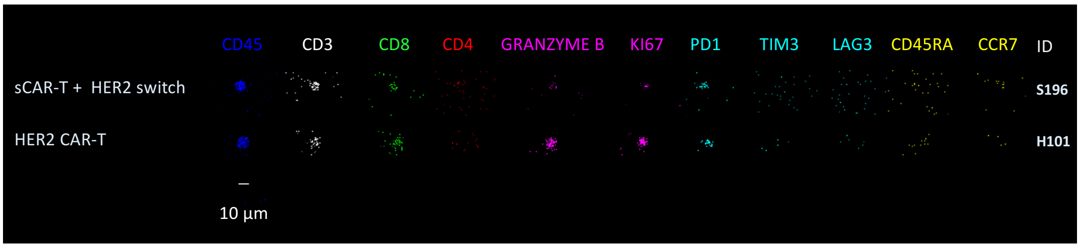

Supplementary figure 6 – Long-term characteristics of CAR-T cells in vivo following treatment of metastatic PDAC tumours. Mouse blood (100 µl) was obtained 41 days following CAR-T cell administration and 21 days after the final switch administration, then pre-sorted for human CD2 to enrich for human T cells. (**A**) Number of sorted human CD2+ T cells detected in the blood of one animal each from the sCAR-T + HER2 switch and sCAR-T + inactive switch groups, respectively. **(B)** Marker expression heatmap for activation, proliferation, exhaustion, and differentiation markers in individual human CAR-T cells detected in the blood of NSG mice (n=3) from the sCAR-T + HER2 switch or HER2 CAR-T cohorts (20e6). Mean signal intensities of each marker in n= 8 representative human $CD3^{+}CD8^{+}$ CAR-T cells is given relative to CD45 expression; green frame indicates markers with significant differences for the two cohorts with $p < 0.05$. (**C**) Marker expression in **(B)** was determined by Hyperion Imaging Mass Cytometry™ multiplex analysis and representative images of individual human CAR-T cells from each treatment group are shown for illustration. Individual CAR-T cells are identified by their ID numbers.

Supplementary figure 7

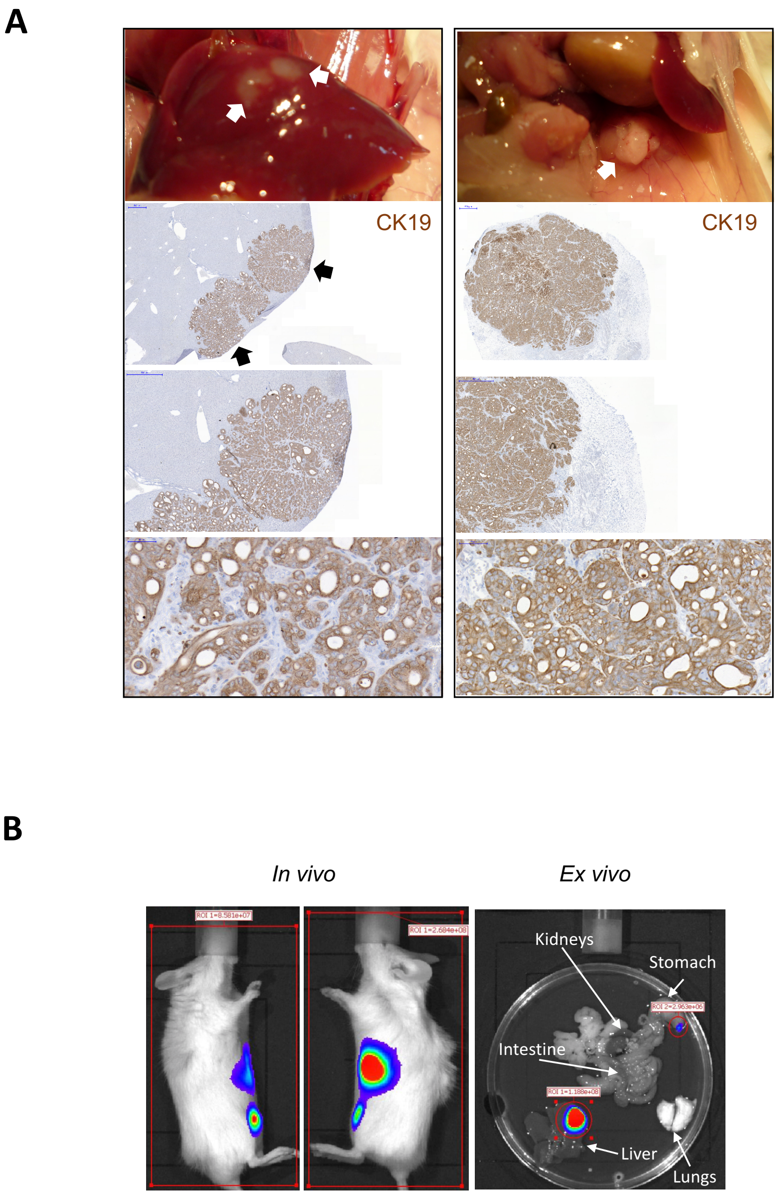

Supplementary figure 7 – CAR-T cell treatment of metastatic PDAC tumours. (**A**) Macroscopic (**top panel)** and microscopic (CK19 staining**; lower panels**) detection of liver metastases (**left**) and local tumours (**right**). **(B)** *In vivo* (**left**) and *ex vivo* (**right**) IVIS images of an NSG animal bearing disseminated PDAC tumours.
